# Identification of microbial markers associated with lung cancer based on multi‐cohort 16 s rRNA analyses: A systematic review and meta‐analysis

**DOI:** 10.1002/cam4.6503

**Published:** 2023-09-07

**Authors:** Wenjie Han, Na Wang, Mengzhen Han, Xiaolin Liu, Tao Sun, Junnan Xu

**Affiliations:** ^1^ Department of Breast Medicine 1 Cancer Hospital of China Medical University, Liaoning Cancer Hospital Shenyang China; ^2^ Department of Pharmacology Cancer Hospital of China Medical University, Liaoning Cancer Hospital Shenyang China; ^3^ Liaoning Kanghui Biotechnology Co., Ltd Shenyang China; ^4^ Key Laboratory of Liaoning Breast Cancer Research Shenyang China; ^5^ Department of Breast Medicine Cancer Hospital of Dalian University of Technology, Liaoning Cancer Hospital Shenyang China

**Keywords:** 16 s rRNA, gut microbiota, lung cancer, lung microbiota, machine learning

## Abstract

**Background:**

The relationship between commensal microbiota and lung cancer (LC) has been studied extensively. However, developing replicable microbiological markers for early LC diagnosis across multiple populations has remained challenging. Current studies are limited to a single region, single LC subtype, and small sample size. Therefore, we aimed to perform the first large‐scale meta‐analysis for identifying micro biomarkers for LC screening by integrating gut and respiratory samples from multiple studies and building a machine‐learning classifier.

**Methods:**

In total, 712 gut and 393 respiratory samples were assessed via 16 s rRNA amplicon sequencing. After identifying the taxa of differential biomarkers, we established random forest models to distinguish between LC populations and normal controls. We validated the robustness and specificity of the model using external cohorts. Moreover, we also used the KEGG database for the predictive analysis of colony‐related functions.

**Results:**

The α and β diversity indices indicated that LC patients' gut microbiota (GM) and lung microbiota (LM) differed significantly from those of the healthy population. Linear discriminant analysis (LDA) of effect size (LEfSe) helped us identify the top‐ranked biomarkers, *Enterococcus*, *Lactobacillus*, and *Escherichia,* in two microbial niches. The area under the curve values of the diagnostic model for the two sites were 0.81 and 0.90, respectively. KEGG enrichment analysis also revealed significant differences in microbiota‐associated functions between cancer‐affected and healthy individuals that were primarily associated with metabolic disturbances.

**Conclusions:**

GM and LM profiles were significantly altered in LC patients, compared to healthy individuals. We identified the taxa of biomarkers at the two loci and constructed accurate diagnostic models. This study demonstrates the effectiveness of LC‐specific microbiological markers in multiple populations and contributes to the early diagnosis and screening of LC.

## 1 INTRODUCTION

Lung cancer (LC) is associated with the fastest‐growing incidence and mortality rates, as compared to other cancers, and represents a severe threat to the health and life of individuals.^1^, ^2^ The early diagnosis rate of LC is about 15%, and most patients present with locally advanced or metastatic disease on diagnosis, which results in a poor prognosis.^3^ Therefore, early screening is pivotal for reducing LC‐related deaths and improving patient survival rates.^4^ However, the acceptance rate of screening is low due to the invasiveness and high cost of current clinical examination methods. Therefore, we urgently need to develop a convenient and cost‐effective strategy for LC diagnosis.^5^

With the advent of precision medicine, artificial intelligence (AI) has come under increased scrutiny.^6^ Machine learning (ML), an important AI technique, shows a compelling performance in clinical aspects such as the diagnosis,^7^ prognosis,^8^, ^9^ and treatment of diseases. In the US, the American Food and Drug Administration has approved AI‐based medical algorithms for diagnosing and evaluating lung nodules and LC.^10^ However, the medical data were based on pathology, radiology, and endoscopy reports. Hence, we attempted to examine whether ML could be used to develop cancer diagnostic models using non‐intrusively obtained samples. The development of early screening methods that are well accepted by the public will accelerate the translation of results obtained using the ML model into real‐world applications.

It had already been reported as early as 2015 that microbial dysbiosis in specific organs could be involved in carcinogenesis. Microbial dysbiosis could affect the host immune system, change the balance between the proliferation and death of host cells, and produce carcinogenic metabolites.^11^ The term “gut‐lung axis” has been coined in recent years to explain the emerging pathogenic links between LC and microbiota.^12^ Subsequently, our focus has shifted to the contributions and implications of microbial species in both microbial niches on LC progression and exacerbation. The lung microbiota (LM) and gut microbiota (GM) of the LC population have been studied in many countries and regions.^13^, ^14^ Zheng et al. conducted a meta‐analysis of 8 LM sequencing studies involving 530 participants. The results indicated that a higher abundance of the *Actinobacteria* and *Firmicutes* phyla occurred in the cancer group compared to the normal control group. At the genus level, there were significant differences in the abundance levels of specific bacteria (e.g., *Prevotella* and *Streptococcus*) in two groups.^15^ Consequently, relevant studies have described the construction of diagnostic models using ML algorithms based on the sequencing data generated using LM DNA samples obtained from LC patients. According to the results reported by Jin et al., the area under the curve (AUC) value of the diagnostic panel built with random forest (RF) regression analysis could increase to 0.882.^16^ Thus, LM is a promising diagnostic biomarker. The gut dysbiosis characteristic of GM has also been extensively reported in LC patients.^17^, ^18^ Modeling studies based on patient GM DNA datasets also represent a popular research topic due to the easy viability of fecal samples and significant differences in the DNA between groups of individuals with and without cancer. Many RF models have been generated for diagnosing LC and predicting subtypes and stages for small‐cohort studies.^19^, ^20^, ^21^, ^22^, ^23^

Although there has been considerable progress in the study of micro biomarkers associated with LC, these studies have been limited to a single region or subtype. No direct evidence indicates the microbiota that would result in the accurate and early detection of LC. There is also no consensus on which genus can be used most effectively for LC screening. To address the above two questions, we conducted a meta‐analysis involving LC populations with varied regions, types, and stages. Studies were conducted with bronchoalveolar lavage fluid (BALF) and bronchial brush samples (as a proxy for LM), and fecal (as a proxy for GM) samples to compare the accuracy of GM and LM markers. We also attempted to elucidate the variations in microbial function to provide theoretical support for further exploring the potential mechanisms by which microorganisms play a role in LC carcinogenesis. Our study demonstrates the validity of LC‐specific micro markers in multiple populations, and our findings would help guide the use of microbes as biomarkers for assessing LC progression and developing targeted therapies.

## 2 METHODS

### 2.1 Search strategy

Two reviewers (WH and YW) from the research team were responsible for conducting systematic literature searches in the Pubmed and Embase databases and performing biological data mining from the Sequence Read Archive (SRA) and European Bioinformatics Institute databases. There were no restrictions on the language or year of publication during retrieval, and information was most recently updated on June 1, 2022. The detailed search strategy is described in Supplementary Section 1.

### 2.2 Inclusion and exclusion criteria

The criteria for selecting articles were as follows^1^: Analysis of GM was performed using fecal matter as the study sample, and it was collected after LC diagnosis and before the patient received treatment.^2^ Representative samples of LM were mainly BALF and bronchial brush samples collected from patients subjected to a bronchoscopy to evaluate lung disease.^3^ The selection of LC patients was not limited by subtype, stage, and smoking history.^4^ The benign group included patients with clinically confirmed benign pulmonary nodules according to disease guidelines.^5^ Normal control individuals had no respiratory symptoms, chest X‐ray abnormalities, or history of lung disease.

The following studies were excluded from the following characteristics^1^: Studies unrelated to the topic^2^; case reports, comments, or review articles^3^; studies in which raw 16srRNA gene sequencing data were not available publicly or could not be grouped clearly^4^; studies involving patients who had received treatment were excluded. Controls with a history of cancer or recent (less than 1 month) use of antibiotics were excluded.

### 2.3 Data extraction and quality assessment

Two reviewers (WH and NW) independently extracted the following information from each study: authors, country, publication year, sample type, sample size, grouping, microbiological assessment method, and NCBI BioProject ID. Two reviewers (WH and MH) independently assessed quality using the ROBIS tool and the Joanna Briggs Institute Critical Appraisal Checklist (Tables S1 and S2; Figure S1). Discrepancies, if any, were discussed with the third reviewer (JX) to reach a consensus.

### 2.4 Data preprocessing

Sequencing and sample data were downloaded from the NCBI SRA project. After the raw FASTQ files were downloaded, they were de‐multiplied, and Vsearch software was used (v2.18.0) to merge raw data, shear primers, and barcodes and filter out low‐quality data.^24^ The generated clean data were merged again, and the feature table and representative sequence were obtained by performing de‐redundancy and denoise processing. Finally, operational taxonomic units (OTUs) with relative abundance levels greater than 1/10000 were selected as the final representative sequence. Based on this sequence, we used QIIME1, a plugin‐based platform, for microbiome analysis and clustering. We classified the relative abundance of the species into five levels: phylum, class, order, family, and genus. Then, the Greengenes (v13.8) database was used to conduct taxonomic annotation.^25^ The generated rarefied data set was used for downstream analyses.

### 2.5 Data analyses

R version 3.6.1 was used for all downstream bioinformatics analysis and data visualization.

#### 2.5.1 Confounder analysis

We used ANOVA‐type analysis to quantify the effects of some confounding factors and disease statuses on a single microbial species. The total variance for a given OTU was compared to the variance attributable to confounders (age, blood glucose level, alcohol consumption history, smoking history, sex, body mass index [BMI], and study), and the variance was attributable to the disease status (cancer‐affected and normal control), like that observed for a linear model. Considering the non‐Gaussian distribution of microbiome abundance data, variance calculations were performed based on ranks.^26^ Confounders with continuous values were converted to categorical data either as quartiles or according to conventional cutoffs for glucose levels to classify individuals as those with hypoglycemia (<3.9 mmol/L), normal glucose levels (3.9–6.0 mmol/L), and hyperglycemia (>6.0 mmol/L).

#### 2.5.2 Diversity analyses

The R package “Vegan” was used for diversity analyses. During α‐diversity analysis, we mainly examined the Chao 1, Shannon, and Simpson indexes. We used the Wilcoxon rank sum test to examine the significance of differences between the two groups. We used an analysis of variance (ANOVA) with an honestly significant difference (HSD) test to examine differences between multiple groups. The R package “Phylose” was used for β‐diversity analysis to perform principal coordinate analysis (PCoA) based on the Bray–Curtis dissimilarity matrix. The analysis of similarities (ANOSIM) was conducted to evaluate the statistical significance, and a box plot was used to visualize the results.

#### 2.5.3 Differential abundance analysis

First, the Wilcoxon rank sum test was used to detect specific species between groups. The cutoff value was a log value >2.0 and *p* < 0.01 in the Wilcoxon rank sum test. The LEfSe was then used to determine the “microbiomeMarker.” Finally, hypothesis testing was performed to assess the significance of the observed differences. The above steps were performed using the “LEfSe” package.

#### 2.5.4 Co‐occurrence and clustering analysis

To further analyze the co‐occurrence of microbiota, we used the R package “co‐occur” to calculate the Spearman correlation between different genera within a group. The R package “psych” was used to screen significant and robust connections (*p*‐value <0.05, | ρ | ≥0.3). The network graph was then visualized using Gephi (v0.9).

#### 2.5.5 Function prediction analysis

PICRUSt (v2.4.2) software was used to predict the functional gene profile based on a previous OUT table. Then, the related gene description information could be annotated from the KEGG database to obtain a functional abundance spectrum. Differential analysis of function was assessed by STAMP software (Welch's *t*‐test; *p* < 0.05).

#### 2.5.6 Random forest model construction and evaluation

We used ML algorithms to construct a classification model and distinguish samples from various groups. First, we split the genus‐level relative abundance dataset into a training set (70%) and a test set (30%), with the training set and test set used for training and performance validation, respectively. We used three algorithms for model training: K‐nearest neighbors (KNN), Support Vector Machine (SVM), and RF to find the most suitable ML method for this study. According to the AUC values obtained with each algorithm and the microbiome‐based classifier used in a previous study,^27^, ^28^ we finally chose the RF R package to construct the predictive model. The hyperparameters ntree = 800 and mtry=$\sqrt{p}$ (number of variables) were set during the analysis, and the other settings were defaulted parameter settings. We identified core biomarkers using “Mean Decrease Accuracy” as the screening index according to importance‐based rankings. Then, we performed 10‐fold cross‐validation of the RF model while obtaining model error values. The receiver operating characteristic curve (ROC) was plotted with the “pROC” package, and the AUC was calculated to evaluate the diagnostic ability of the model. The ML algorithm selection process and tuning hyperparameters are described in Supplementary Section 2.

To verify the applicability of the model in different contexts, we performed study‐to‐study transfer validation and leave‐one‐dataset‐out (LODO) validation. During study‐to‐study transfer validation, one item was set in the test set, and another was set in the training set (LODO validation: the remaining items are confluent as the training set). Then, AUC cross‐tabulations were performed based on the screened core biomarker.^29^ In addition to internal validation, independent studies and individuals with lung disease need to be involved during external validation to demonstrate the reproducibility and specificity of the model. For the 16 s sequence type, external data could be applied directly to the test dataset, and predictions were summarized to the ROC values. For whole genome sequencing (WGS) data, species consistent with the results of the 16 s model were screened, and their abundance levels were substituted into the 16 s model to investigate the AUC value.

## 3 RESULTS

### 3.1 Overview of the study cohort characteristics in the meta‐analysis

Based on the inclusion criteria, 195 articles retrieved from PubMed.gov and EMBASE were critically reviewed. Figure S2 shows the selection process for this study. A total of seven studies met our inclusion criteria. These studies involved populations from China, South Korea, the US, and six European countries (UK, Germany, Italy, Poland, Hungary, and Portugal). We collected 712 gut samples (fecal) and 393 lung samples (343 BALF and 50 bronchial brush samples), including samples for the validation cohort. Detailed information regarding all the cohorts used in this meta‐analysis is listed in Table 1. The sample processing procedures and analytical methodology of each study are summarized in Table S3 and Table S4, respectively.

**TABLE 1:** Characteristics of the included datasets in a systematic review.

Study	First author	Year	Population	Sample source	Sample numbers	Accession number	16 s region	Sequencing platform
Study 1	Hui Lu	2021	China, Wuhan	Fecal	114	PRJNA576323	V3‐V4	Illumina MiSeq
Study 2	Feng Zhao	2021	China, Hangzhou	Fecal	79	PRJNA736821	V3‐V4	Illumina MiSeq
Study 3	Se‐Hoon Lee	2021	Korea, Seoul	Fecal	234	PRJEB26531	V3‐V4	Illumina MiSeq
Study 4	He Zhuang	2019	China, Harbin	Fecal	60	PRJNA507734	V3‐V4	Illumina MiSeq
Study 5	Susana Seixas	2021	Portugal, Porto	BALF	56	PRJNA742244	V4	Illumina MiSeq
Study 6	Jun‐Chieh J. Tsay	2018	USA， New York	BALF	52	PRJNA397867	V4	Illumina MiSeq
Study 7	Mohammadali Yavari Ramsheh	2021	EUR	Bronchial brushes	50	PRJNA632472	V4 and v5	Illumina MiSeq
Validation Study 1	Yoshitaro Heshiki	2020	USA， Phoenix, AZ	Fecal	26*	PRJNA494824	NA	Illumina MiSeq
Validation Study 2	Demin Cao	2021	China， Beijing	Fecal	51	PRJNA715947	V3‐V4	Illumina HiSeq
Validation Study 3	Lesa Begley	2018	USA, Michigan	Fecal	59	PRJNA474717	V4	Illumina MiSeq
Validation Study 4	Susana Seixas	2021	Portugal, Porto	BALF	17	PRJNA742244	V4	Illumina MiSeq
Validation Study 5	Shashank Gupta	2021	India, Delhi	BALF	35	PRJNA512576	V3‐V4	Illumina MiSeq
Validation Study 6	Rachele Invernizzi	2020	UK, London	BALF	183	PRJNA609242	V4	Illumina MiSeq

*The taxonomic profiles for a total of 16 stool samples from the Human Microbiome Project (HMP), as provided by MetaPhlAn2 (http://segatalab.cibio.unitn.it/tools/metaphlan2/), were used as a healthy control in the taxa comparison.

Abbreviations: BALF, bronchoalveolar lavage fluid; EUR, Leicester, Manchester, and Coventry [UK]; Munich, Marburg, and Freiburg [Germany]; Ferrara [Italy]; Warsaw [Poland]; and Budapest [Hungary]); NA, not available.

### 3.2 Confounder analysis of the microbiomes associated with LC

**FIGURE 1:**
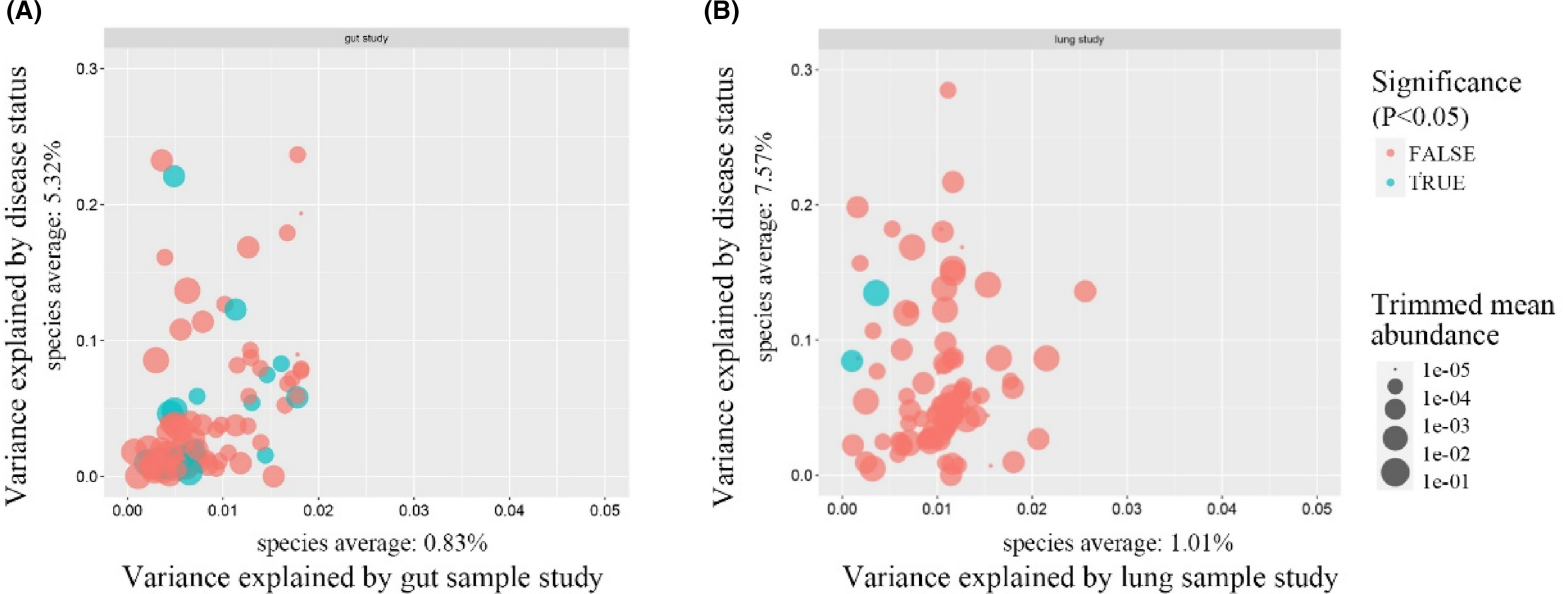
Variance explained by disease status (LC vs. Normal) is plotted against variance explained by the study of gut (A) and study of lung (B) effects for individual microbial species. The significantly differential OTUs are colored in blue, and P values were from the two‐way ANOVA test. The abundance of each genus is plotted proportionally to the dot size. LC, lung cancer; OTUs, operational taxonomic units; ANOVA, Analysis of Variance.

Due to the technical and biological differences among the included studies, we first quantified the influence of the confounders associated with the studies on microbiome composition. The results showed that the variance attributable to the factor “study” was more significant than that attributable to disease status and other confounding factors, which had the highest impact on microbial composition (Figure 1, Figure S3). Therefore, we used “study” as a blocking factor and used a two‐sided blocked Wilcoxon rank sum test to adjust for batch effects. The differential OTUs with the most negligible impact from the “study” were selected for subsequent analysis.

### 3.3 Alteration of the microbiota of the lung cancer population

#### 3.3.1 Characteristic changes in the gut microbiota

**FIGURE 2:**
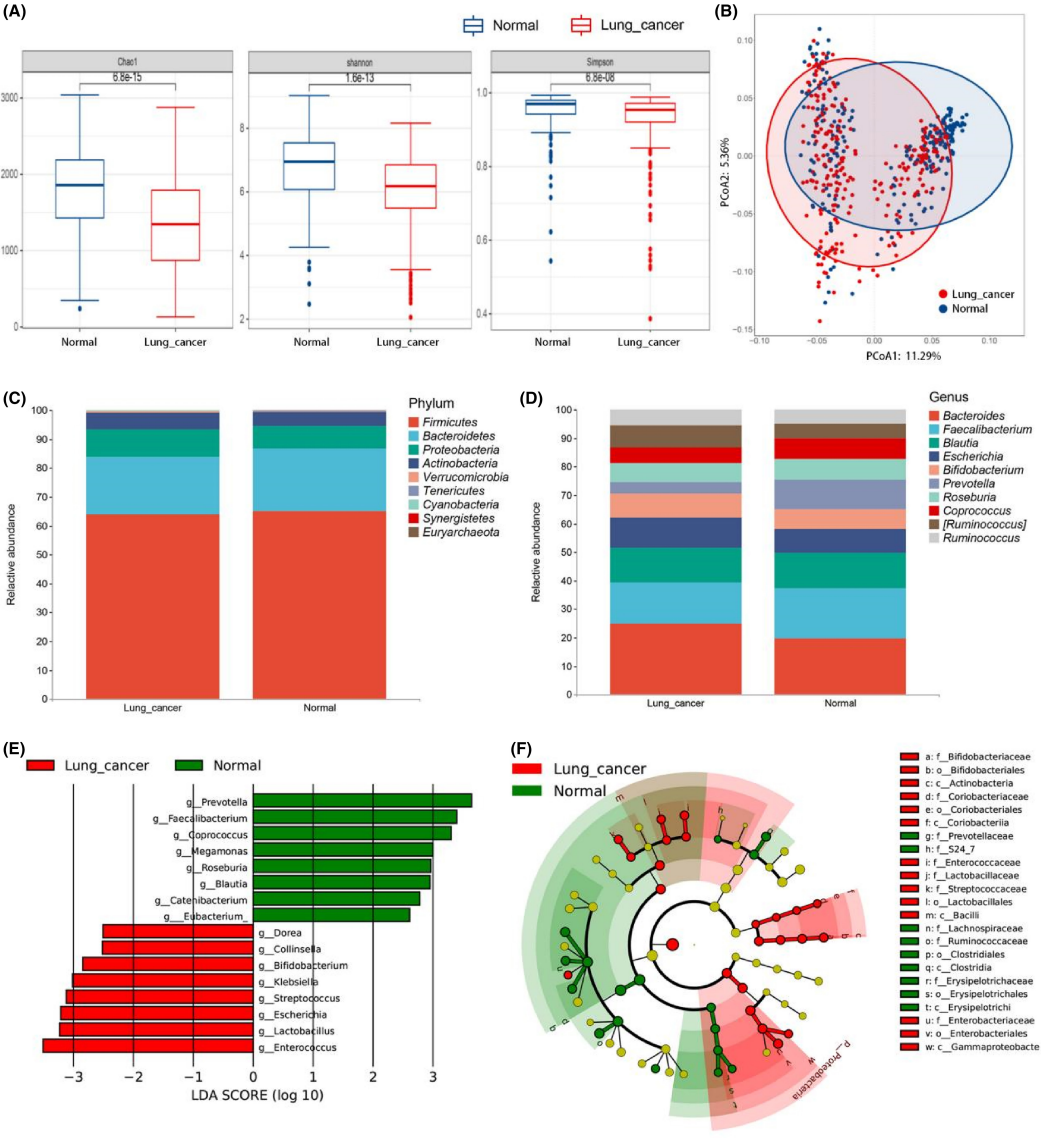
Alterations of gut microbiota composition in lung cancer. (A) Differences in α diversity between Lung cancer and Normal group based on the standardized OTUs table, Chao 1, Shannon, and Simpson indices. (B) The β diversity was evaluated by PCoA based on Bray Curtis distance, which shows the GM composition was different among groups. (C) Phylum‐level taxonomic profiles of LC patients and normal individuals. (D) Genus‐level taxonomic profiles for the two groups of samples. (E) Histogram of the distribution of LDA values for LEfSe analysis of two groups. (F) Evolutionary map of species branching for LEfSe analysis of GM in two groups. OTUs, operational taxonomic units; PCoA, principal coordinate analysis; GM, gut microbiota; LC, lung cancer; LDA, Linear discriminant analysis; LEfSe, Linear discriminant analysis Effect Size.

In Figure 2A, the indices for the LC group were always slightly lower than those for the normal group, which was in accordance with previous findings.^19^ This indicates that the decrease in species diversity is one of the manifestations of gut dysbiosis in LC patients. We evaluated the differences and similarities between the two groups, that is, we conducted a β diversity analysis (Figure 2B). The results of PCoA analysis showed that there was a notable level of variability between the two groups when PCoA 1 (11.29%) and PCoA 2 (5.36%) were used as the abscissa and ordinate, respectively (*p* = 0.001). ANOSIM analysis also showed that there were significant differences between the LC and normal groups (R > 0, *p* = 0.001) (Figure S4A). In conclusion, our meta‐analysis revealed significant differences in the species diversity and composition of the GM between LC patients and controls.

We annotated the species of all OTUs and identified 11 phyla, 17 orders, 21 families, 42 families, and 89 genera. At the phylum level, *Firmicutes* were the prominent GM members in the LC group, followed by *Bacteroidetes*, *Proteobacteria*, and *Actinobacteria*. The dominant phyla in the normal group were similar to those in previous studies and did not differ significantly from the LC group (Figure 2C). However, the differences were more evident at the genus level. In the LC group, the abundance levels of *Bacteroides* and *Escherichia* increased significantly, while those of *Prevotella* and *Coprococcus* decreased significantly (Figure 2D). We performed multi‐LEfSe to identify statistically significant biomarkers. The results showed that species‐related differences could mainly be attributable to the presence of *Prevotella*, *Faecalibacterium*, and *Enterococcus* (Figure 2E). Some bacterial taxonomic clades were significantly different in the LC and control groups (log10 [LDA score] > 2) (Table S5). Eight genera, including *Enterococcus*, *Lactobacillus*, *Escherichia*, and *Streptococcus*, were markedly enriched in the LC group. These might represent important markers for the early screening of LC. Then, we analyzed the evolutionary relationships of the GM species. The evolutionary map of species branching during LEfSe analysis has been shown in Figure 2F. The data showed that the dominant flora of the two groups was significantly different and was synchronized with the histogram showing the distribution of LDA values.

#### 3.3.2 Alterations in the composition of lung microbiota in lung cancer patients

In the meta‐analysis, samples were obtained from the involved airway (the lung nodule segment) or the uninvolved airway (usually in the lobe contralateral to the suspicious nodule). To ensure the accuracy of the results, we performed PCoA analysis for these samples (Figure S5). The results showed no significant differences in the microbial composition of the airways involved and uninvolved with LC, and the same results were also observed for benign samples. Therefore, we combined all lower respiratory samples for performing the subsequent analyses.

**FIGURE 3:**
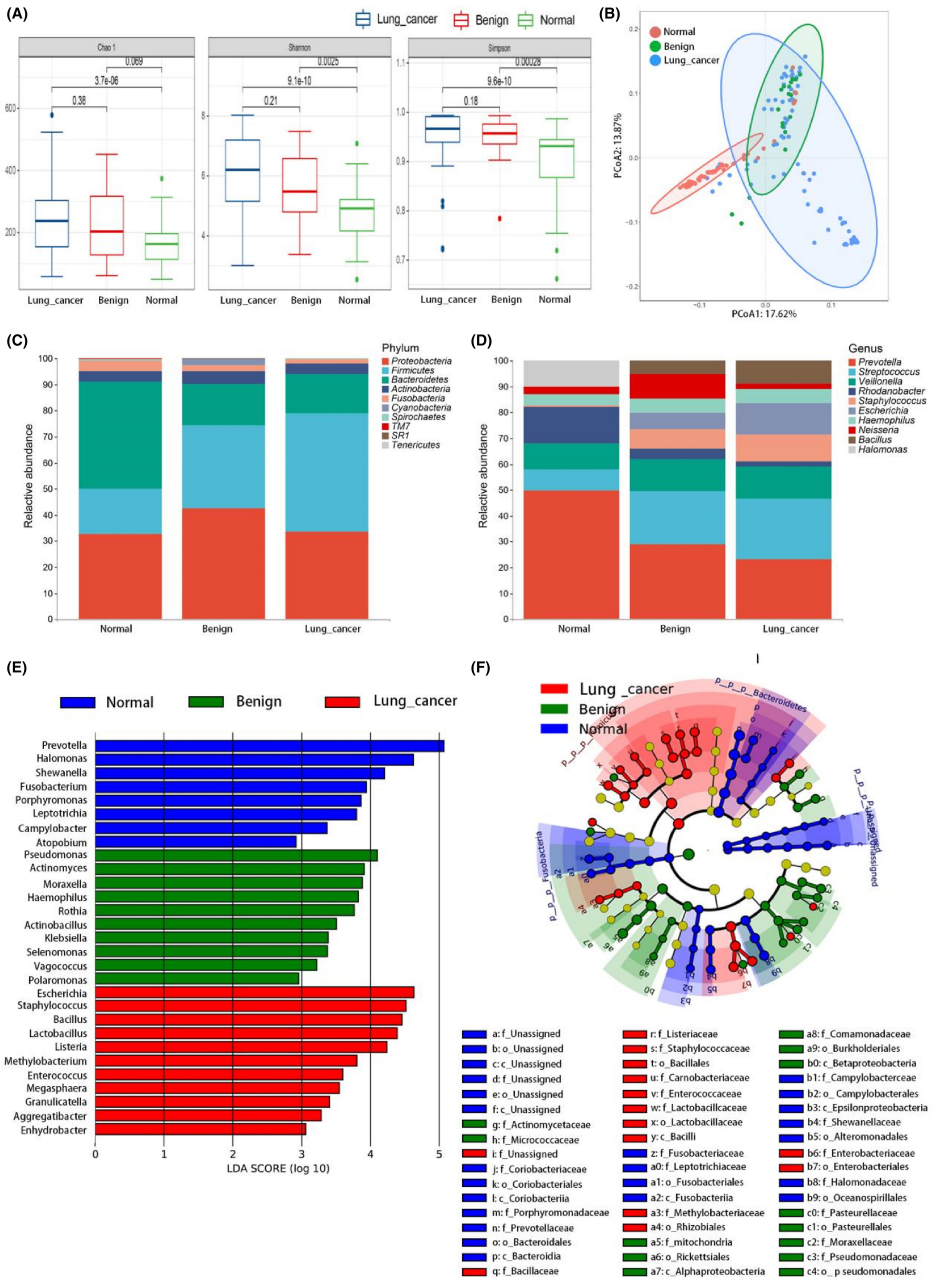
Microbial composition and difference analysis of lung microbiota in the Lung cancer group, Benign group, and Normal group. (A) Comparisons of α‐diversity between different groups. α diversity, measured with the Chao 1, Shannon, and Simpson index, was computed with all OTUs in all samples. (B) β diversity as shown by PCoA of Bray Curtis distances. (C)Taxonomic composition at phylum level in lung samples. (D) Taxonomic composition at genus level in lung samples. (E) Differential taxa identified by LEfSe with LDA values of 2. Taxa enriched in different groups are displayed by color indicated in the key (red indicating taxa abundant in the LC group, blue in the Normal group, and green in the Benign group). (F) Cladogram showing the phylogenetic distribution of microbiota associated with three groups. OTUs, operational taxonomic units; PCoA, principal coordinate analysis; LEfSe, Linear discriminant analysis Effect Size; LDA, Linear discriminant analysis; LC, lung cancer.

We also performed a diversity analysis, and Figure 3A shows that the α diversity gradually increased with disease development. The Simpson index is the most notable index that showed value changes. PCoA analysis showed more pronounced intergroup differences in the LM than in the GM (Figure 3B). ANOSIM analysis also confirmed these results (Figure S4B). The species mainly belonged to dominant phyla such as *Proteobacteria*, *Firmicutes*, and *Bacteroidetes*, similar to those in the gut but present at slightly different proportions (Figure 3C). At the genus level, it can be seen that the dominant genera were significantly different between the normal control group and the disease group (LC and benign). *Prevotella* and *Rhodanobacter* levels were reduced considerably in the disease population (Figure 3D).

We also performed LEfSe to reveal potential differential microbial taxa among the three groups (Figure 3E, F). At the genus level, *Escherichia*, *Staphylococcus*, and *Bacillus* were representative markers of the LC group, and *Prevotella* and *Pseudomonas* characterized the normal control and benign groups, respectively (Table S6).

#### 3.3.3 Association and differences between GM and LM in lung cancer

**FIGURE 4:**
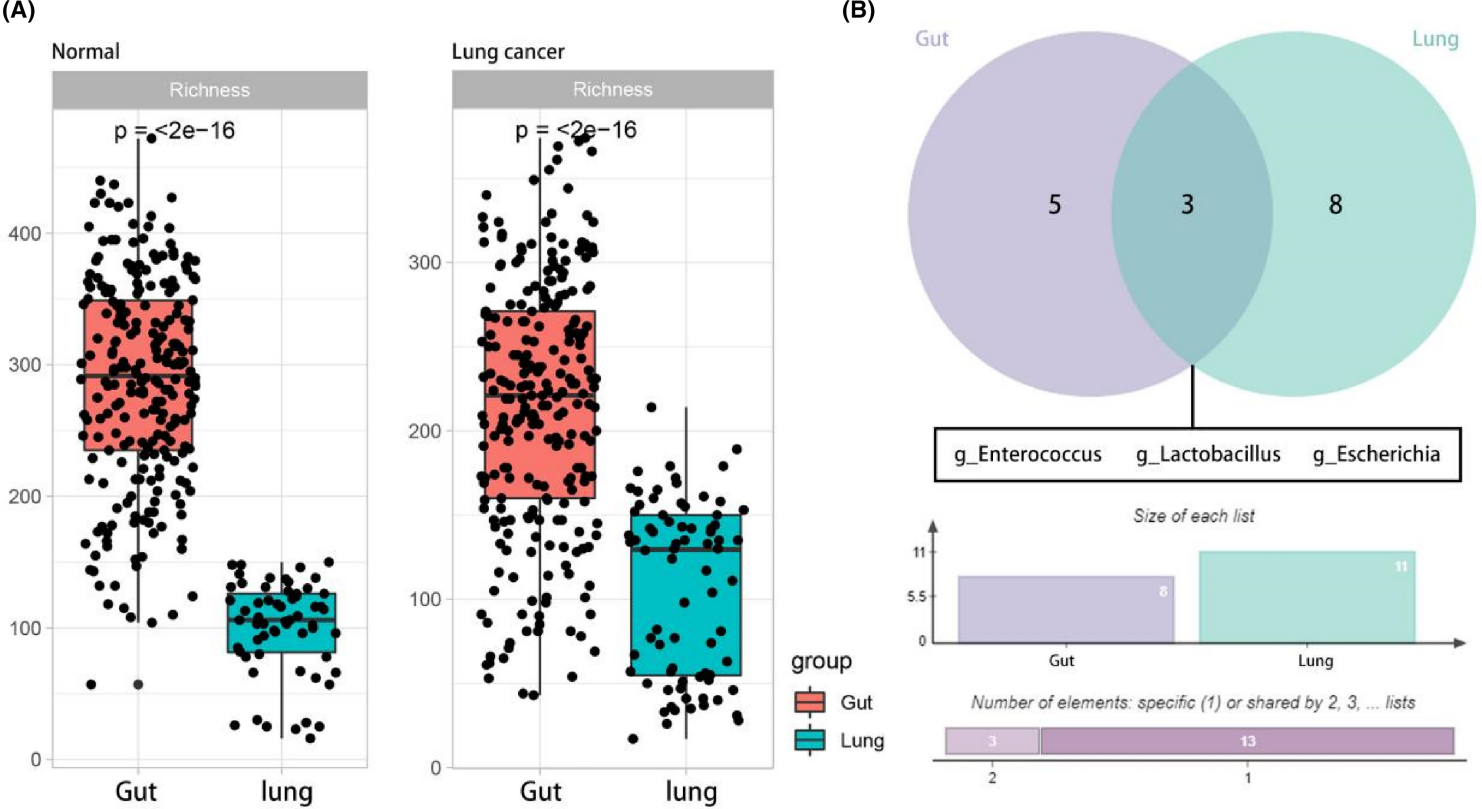
Differences and relationships between gut microbiota and lung microbiota. (A) Gut and lung microbiota differed significantly in terms of α diversity. Comparisons of Richness index (Normal group, left; Lung cancer group, right). (B) Overlap of gut and lung biomarkers of lung cancer in Venn diagram.

As shown in Figure 4A, the microbial diversity was significantly higher in the gut than in the lung, in the normal control and LC groups. Interestingly, we found an overlap between the biomarkers of the LC group at two loci and identified three shared biomarkers: *Enterococcus*, *Lactobacillus*, and *Escherichia* (Figure 4B). All three genera may play essential roles at the two sites of tumorigenesis.

### 3.4 Co‐occurrence network analysis of microbiota

**FIGURE 5:**
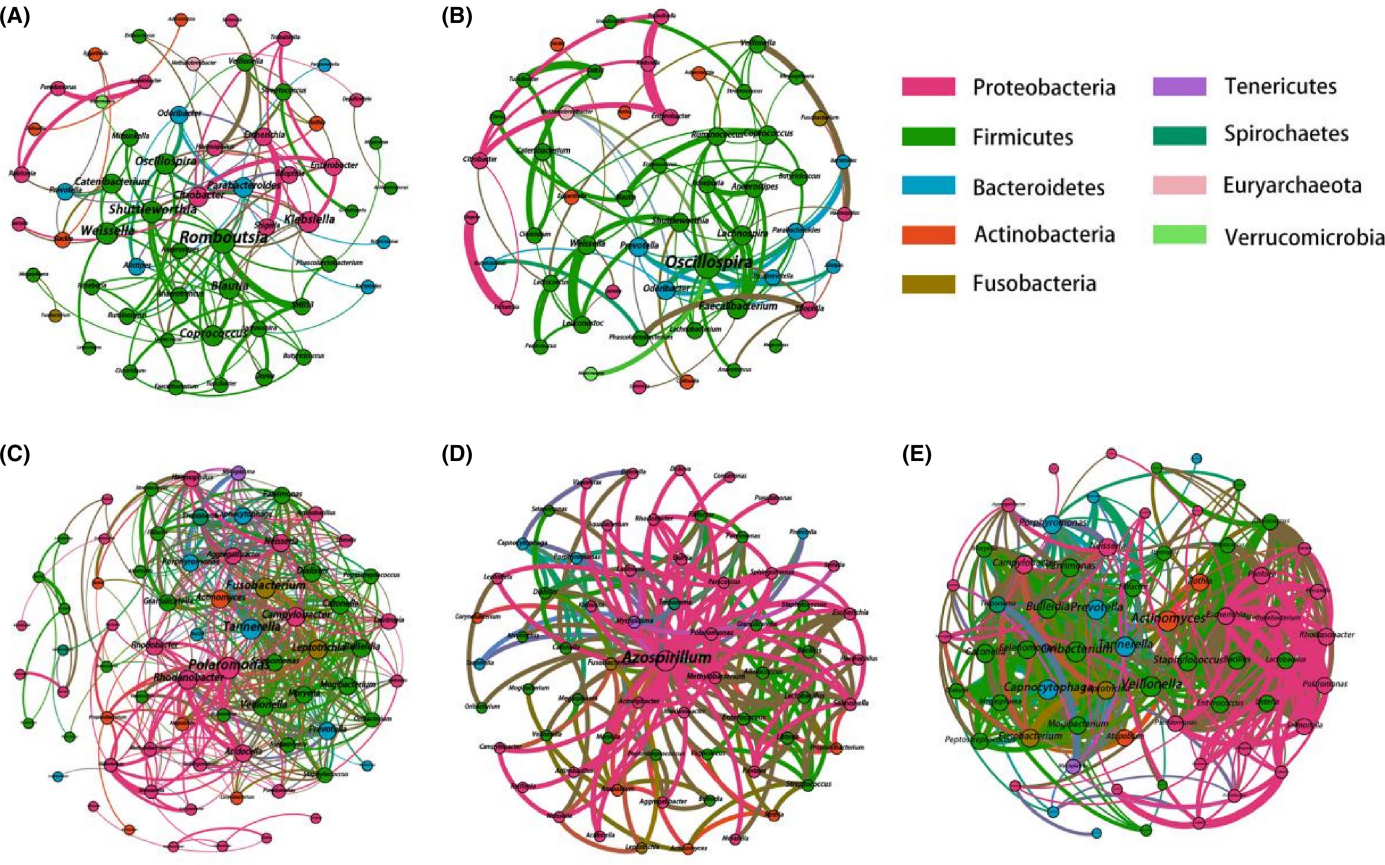
Co‐occurrence network of the microbiota in the different groups. The correlation coefficient was calculated with the Spearman rank correlation test (| *ρ* | ≥ 0.3). (A) Correlation networks in Normal group of gut microbiota. (B) Correlation networks in Lung cancer group of gut microbiota. (C) Correlation networks in Normal group of lung microbiota. (D) Correlation networks in Benign group of lung microbiota. (E) Correlation networks in Lung cancer group of lung microbiota. Each circle represents the average relative abundance of a microbial species in that state. Node sizes are scaled according to their degrees of connection. The thickness of the line represents the strength of the relationship.

In order to thoroughly understand the correlation among microorganisms in each group and the strength of their interactions, we constructed a co‐occurrence network based on previous results. The number of nodes and association density of the GM group (Figure 5A, B) were significantly lower than those of the LM group (Figure 5C–E), indicating that the relationship with LM is closer than that with GM and involved a more comprehensive range of bacteria.

With regard to LM, the LC group (Figure 5E) had more edges and higher network densities than the normal control (Figure 5C) and benign groups (Figure 5D). This suggests that disease occurrence enhances the original interactions between flora. The same phenomenon can be observed in GM, and the correlation curve was thickened in the LC group (Figure 5B). In addition, the size of the nodes also revealed that some essential bacterial genera potentially acted as key hubs in the community. For example, *Oscillospira* was more closely associated with other genera in the gut LC group. Among the lung LC group, *Veillonella*, *Actinomyces*, and *Oribacterium* had higher centrality levels.

Overall, interaction‐based relationships were observed to a more significant extent in *Proteobacteria* and *Firmicutes*. *Firmicutes* contain many butyrate‐producing bacteria, and many studies have revealed that butyrate has antitumor properties.^30^, ^31^ This indicates that some strains of *Firmicutes* are likely to be involved in antagonism and competition with pathogenic bacteria, leading to more complex networks in the community, which also explains the coarser correlation curves and a stronger degree of clustering of *Firmicutes* species in the LC group.

### 3.5 Diagnostic models for lung cancer based on different micro‐ecological loci

#### 3.5.1 Construction of microbial classification models

**FIGURE 6:**
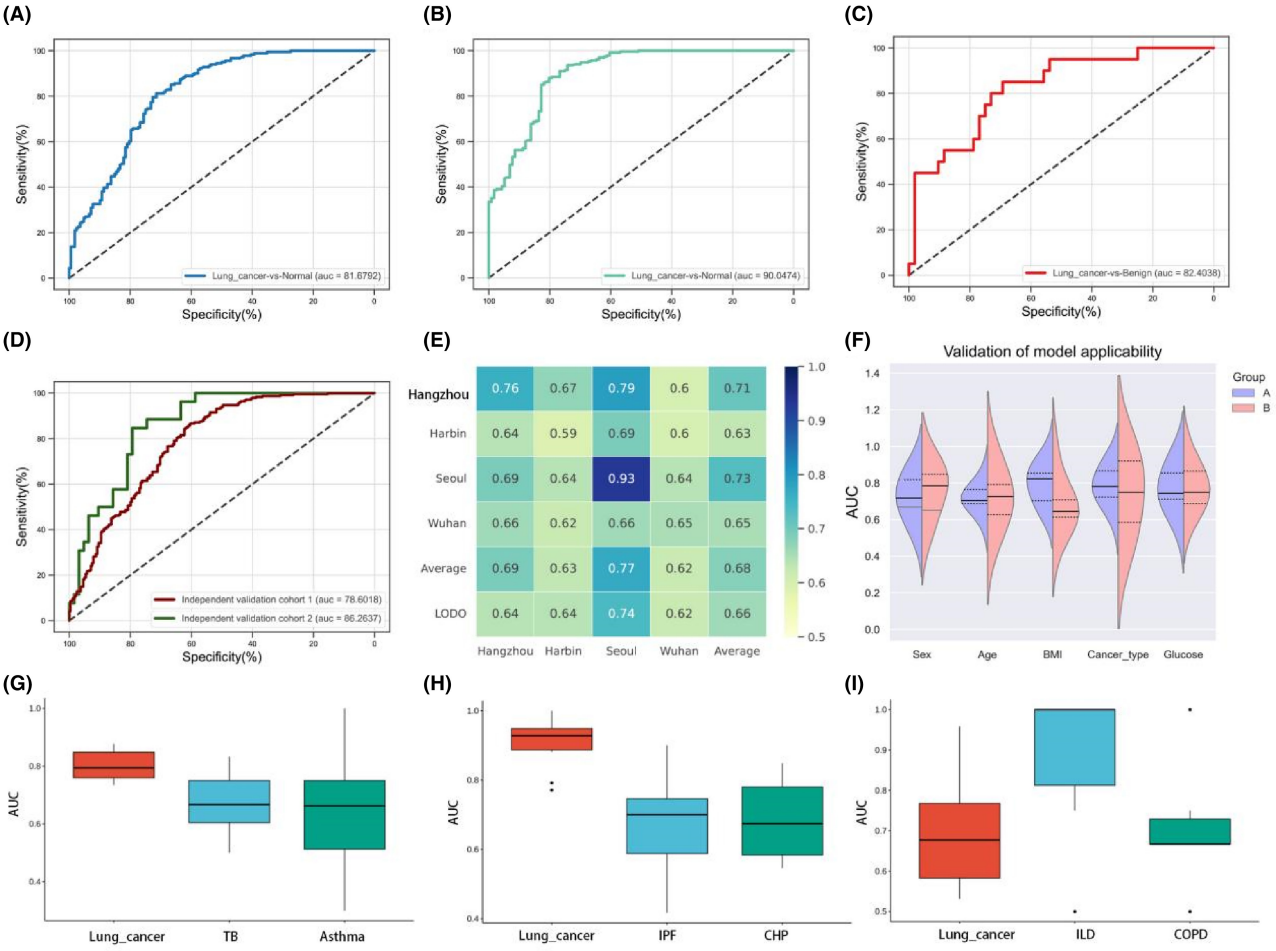
Construction and validation of a diagnostic model based on gut and lung‐specific microbiota. (A)The ROC of LC versus Normal group classification model based on the GM. (B) The ROC of the LC versus Normal group classification model based on the LM. (C) The ROC of the LC versus Benign group classification model based on the LM. (D) Validation of GM classification model in two independent cohorts. (E) Cross‐prediction matrices were constructed using the study‐to‐study transfer validation and LODO validation values for gut classifiers. The value on the diagonal is the cross‐validation result of a single study, and the off‐diagonal is the cross‐validation result between cohorts. (F) Validation of clinical applicability of GM classification model. A and B are different groups with the same clinical parameters, Sex (A/B, Male/Female); Age (A/B, age>60/age ≤ 60); BMI (A/B, BMI≤24/BMI>24); Cancer type (A/B, LUAD/SCC); Glucose (A/B, 70 < Glu < 120/Glu≥120). (G) The specificity of gut predictive models. Lung‐related diseases were used to validate the specificity of the GM classification model: TB (*n* = 58) versus Normal (*n* = 22) model, asthma (*n* = 45) versus Normal (*n* = 14) model. (H) Specific Validation of the LC versus Normal group model based on the LM: IPF (*n* = 45) versus Normal (*n* = 28) model, CHP (*n* = 110) versus Normal (*n* = 28) model. (I) Specific validation of the LC versus Benign group model: ILD (*n* = 18) versus Benign (*n* = 15) model, COPD (*n* = 34) versus Benign (*n* = 15) model. ROC, receiver operating characteristic curve; LC, lung cancer; GM, gut microbiota; LM, lung microbiota; LODO, leave‐one‐dataset‐out; BMI, body mass index; LUAD, Lung Adenocarcinoma; SCC, squamous carcinoma; Glu, glucose; TB, tuberculosis; IPF, idiopathic pulmonary fibrosis; CHP, Chronic hypersensitivity pneumonitis; ILD, interstitial lung disease; COPD, chronic obstructive pulmonary disease.

The AUC of the GM‐based classifier model was 0.81 in the LC group versus the normal control group, based on the 36 feature variables **(**Figure 6A **)**. The LM‐based classifier model had a higher ability to distinguish between individuals with and without cancer based on the 26 feature variables (AUC = 0.90, Figure 6B **)**. We also found that LM had good diagnostic capabilities and could effectively distinguish the LC group from the benign group based on the seven feature variables (AUC = 0.81, Figure 6C **)**. The comprehensive performance indicators of three RF models on the testing dataset are shown in Table 2. Our results show that both sites of microbial markers have an excellent diagnostic value, and the performance of LM when used for disease prediction was better than that of GM.

**TABLE 2:** Performance indicators of three models on the testing dataset.

Model	Group	Specificity	Sensitivity	Accuracy	Kappa coefficient
Gut	Lung cancer vs. Normal	0.637	0.961	0.805	0.605
Lung	Lung cancer vs. Normal	0.773	0.954	0.882	0.832
Lung	Lung cancer vs. Benign	0.550	0.980	0.861	0.605

#### 3.5.2 Validation of the robustness of the microbial classifier

We performed study‐to‐study transfer and LODO validation within the included projects to test whether these two classification models are universal and robust across multiple studies. In the GM classifier, the AUC values for study‐to‐study transfer validation ranged from 0.59–0.93, with a mean of 0.66 (Figure 6E). Notably, a relatively higher testing value (AUC = 0.93, mean 0.73) was observed for the Seoul group as a training set, which can be explained by the relatively large sample size of its dataset. The results of the LODO analysis showed that the AUC of the gut microbial classifier ranged from 0.62–0.74 (average AUC = 0.66). To confirm the results of 16 s rRNA gene sequencing, we included two additional independent cohorts for external Validation (Figure 6D). The RF model of the independent cohort resulted in AUC values of 0.86 and 0.78, respectively. In addition, we examined the clinical applicability of the GM model. The analysis showed that the BMI significantly affected the model results, and individuals with high BMIs had the best model predictions (Figure 6F).

#### 3.5.3 Assessing the specificity of predictive models

In order to reduce the occurrence of false positive results in clinical diagnosis, it is necessary to further confirm the specificity of the predictive model. In this analysis, we considered six non‐LC diseases, including tuberculosis (TB), asthma, idiopathic pulmonary fibrosis (IPF), chronic hypersensitivity pneumonitis (CHP), interstitial lung disease (ILD), and chronic obstructive pulmonary disease (COPD). For GM models, as seen from the box plot for AUC, the AUC values for the non‐LC disease models were significantly lower than for the LC model **(**Figure 6G **)**. The LC versus normal model based on LM also showed good specificity (Figure 6H), while the LC versus Benign model assessment was poor (Figure 6I). These results emphasize that the markers used to distinguish between LC patients and normal controls are specific and exclusive, without interference from associated lung disease. However, it is insufficient to differentiate among lung disease types while attempting to perform a more precise LM‐based analysis. It is evident that individuals with lung diseases have remarkably similar states of LM dysbiosis and may have some common pathogenic bacteria.

### 3.6 Altered microbial functions in lung cancer

**FIGURE 7:**
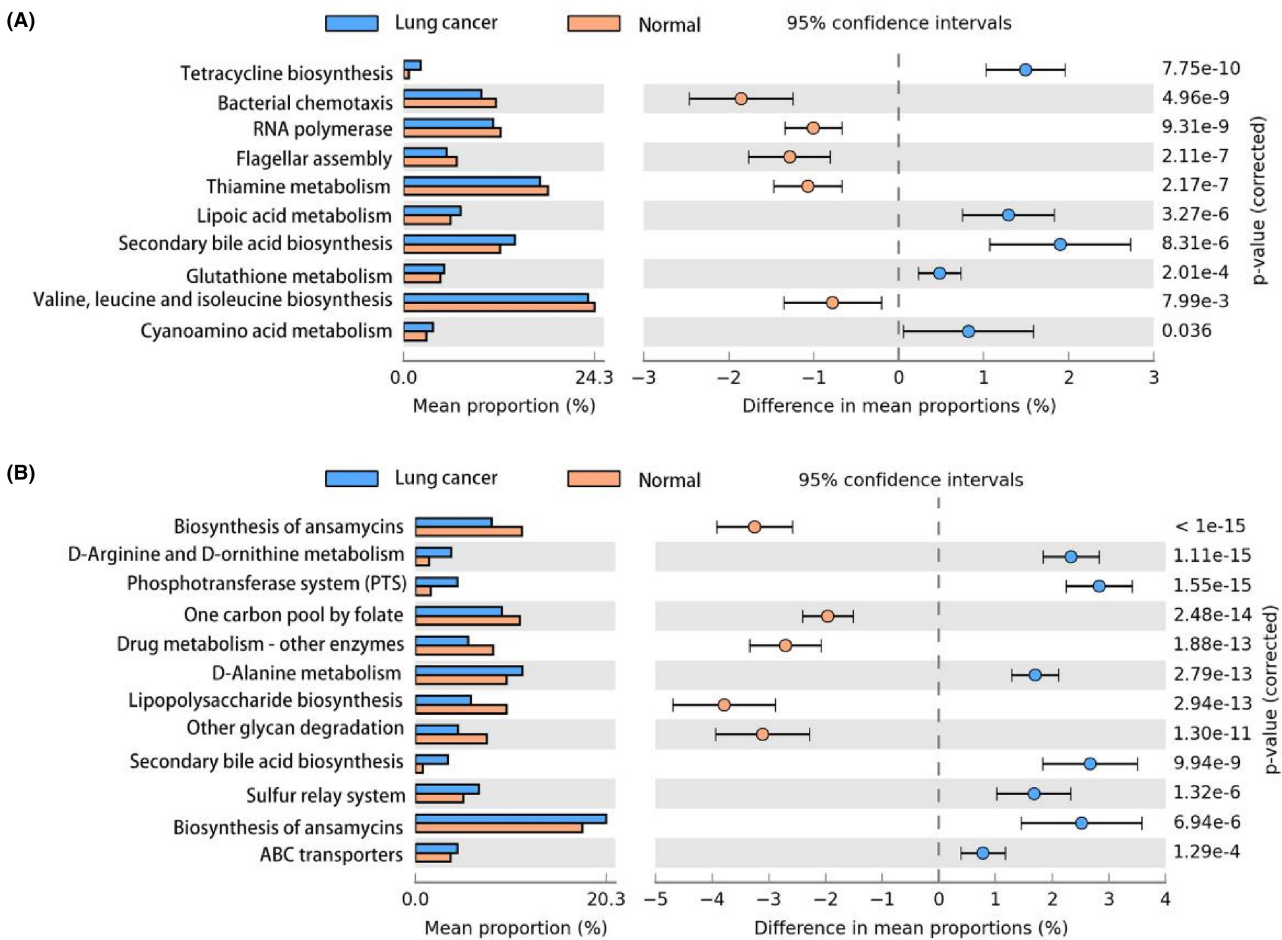
Altered functions in the gut (A) and lung (B) microbial communities. KEGG pathway with significant differences between the Lung cancer group and the Normal group. KEGG, Kyoto Encyclopedia of Genes and Genomes.

Overall, the functional differences in GM **(**Figure 7A **)** between LC patients and controls were less significant than those with LM **(**Figure 7B **)**. This may be attributable to the location of LM being close to the lesion and the considerable effect of the pathological lung environment.

We identified 50 and 75 marker pathways in the gut and lung, respectively. Among the GM marker pathways, we found 21 pathways, which included pathways for secondary bile acid biosynthesis, tetracycline biosynthesis, and lipoic acid metabolism, which were upregulated in the LC group (Table S7). In contrast, functions associated with bacterial chemotaxis and flagellar assembly were decreased. Among the 75 marker pathways in the LM, we found that pathways related to the phosphotransferase system and D‐arginine and D‐ornithine metabolism were enriched in the LC group (Table S8). The pathways related to lipopolysaccharide biosynthesis were downregulated in the LC group. Finally, we tried to identify the functional differences and relationships between the two sites and found seven common upregulated pathways (Figure S6A). These pathways may play a key role in LC carcinogenesis. Although the above results were obtained from PICRUSt 2.0, the results suggest that different degrees of metabolic reprogramming might occur at different sites in the microbiotas during LC progression. And the prediction function of WGS data also confirmed the accuracy of some functions of 16 s prediction (Figure S6B).

## 4 DISCUSSION

This study comprehensively assessed the capability of LM and GM for early LC detection. Our results show that GM and LM exhibit good predictive ability for LC screening. In addition, we found that LM also exhibited good performance in distinguishing between benign lung disorders and LC. Still, a subsequent specificity‐related validation showed that the model was susceptible to interference from other lung diseases. Finally, we constructed two microbial classification models to screen the LC and normal groups (AUC_gut_ = 0.81; AUC_lung_ = 0.90). Considering the non‐invasiveness, convenience of use, and cost‐effectiveness, we believe that the GM model is more suitable for developing a new method for early screening. We also conducted a multi‐level validation process for the gut model, and the results conclusively proved the robustness of the classifier. The results provide evidence for the feasibility of the use of GM for the non‐invasive diagnosis of LC.

The predictive performance of GM as an independent diagnostic tool has been demonstrated in more than 20 diseases.^32^ For LC, the diagnostic ability of the GM model constructed by Wang et al. (AUC = 0.85) was slightly higher than that of our model (AUC = 0.81).^19^ Through methodological comparison, we found that their study used logistic regression analysis. The linear simulation associated with regression analysis is simpler and faster to run, but confidence interval coverage may deteriorate at higher levels of data complexity.^33^ As for the meta‐data, the RF algorithm can effectively evaluate the accuracy of various features during the classification process and help us obtain reliable results for missing values.^34^ Importantly, research by Qi et al. showed that RF had superior performance compared to other multi‐class classifiers (KNN, SVM, graph convolutional neural network, and multi‐layer perceptron) when GM data was used for training.^27^ In addition, in studies on constructing classifiers for colorectal cancer, Crohn's disease, obesity, and pancreatic cancer, RF showed the best discriminative performance.^35^, ^36^, ^37^, ^38^ Therefore, we believe that the RF model can solve our problems more effectively. In addition, Lu et al. and Lim et al. also adopted the RF algorithm, and the prediction accuracy was >0.7.^20^, ^23^ Again, the feasibility of this algorithm is demonstrated. However, very few of these classification rules have been tested in independent studies. In other studies that examined the same problem/data, the included population was limited to a single LC subtype or the local area, which may not be a good explanation of commonality representative of multiple populations with LC.^19^, ^20^

We included all subtypes of LC and several regions in our study, because of which our findings have more substantial applicability and repeatability. For gut samples, the dominant phylum and genus identified by our meta‐analysis were in accordance with those identified in previous studies; the proportion alone is slightly different.^19^ This may be attributable to inevitable clinical factors, such as the sample types, regional differences, and analytical tools. We performed LEfSe analysis to identify the taxa of gut biomarkers between the two groups. The results showed that *Enterococcus* was the most common marker. Notably, it was also screened as a biomarker during LM analysis. Zhuang et al. reached the same conclusion in their study.^39^ Current studies on *Enterococcus* have focused on exploring its association with disease prognosis. In a WGS study of GM, *Enterococcus casseliflavus* was shown to serve as a biomarker of response to chemotherapy in LC patients.^40^ In addition, an article published in Science also noted that the occurrence of *Enterococcus prophage* in the gut of LC patients is significantly associated with the long‐term benefits of PD‐1 blockade therapy.^41^ However, it has also been shown that *Enterococcus* has a growth‐promoting effect on A549 cells (a non‐small cell lung cancer cell) while altering its stiffness.^42^ Thus, it is essential to be aware of the potential harm caused by *Enterococcus* as a cancer promoter and take advantage of its benefits in adjuvant therapy, in order to maximize the benefits of the microorganism to the host.

In addition to GM, other types of microbiota also showed powerful classification ability. For example, Lu et al. used data from sputum microbiota as the original data for modeling (AUC = 0.75).^20^ However, microorganisms are not the best classifying factor for sputum samples, and *microRNA‐21* and *TMEM196*, isolated from the sputum, could provide more accurate diagnostic results.^43^, ^44^ In another study, *Veillonella* and *Capnocytophaga* in the saliva could yield a receiver operating characteristic value of 0.86.^45^ However, because sputum and saliva are susceptible to contamination with oral microbiota at the time of collection, the accuracy of the findings is questionable.^46^ Theoretically, the LM is an ideal sample that could be used to study LC, as it is the closest to the lesion in a spatial location. It reflects the close relationship between the microbiome and the disease most accurately. Previous studies have demonstrated that BALF samples could reflect the LC tissue microbiota more effectively than sputum samples.^47^ Bello et al. showed that the development of the model also proves the high diagnostic value of microorganisms identified during a bronchial biopsy (AUC = 0.89).^48^ Although LM can help obtain a definite histological diagnosis of LC, it may cause bleeding, resulting in an invasive injury and extreme discomfort during sampling.^49^ Hence, this sample type is unsuitable for screening the general population and is more appropriate for individuals known to have lung disease.

In our lung sample study, the lung microbiome was more similar in patients with lung disease and lung cancer, and Jin et al. obtained a conclusion consistent with ours.^16^ Furthermore, the diagnostic model they developed had an AUC value of 0.88. It is important to note that they used WGS data. The WGS data are more accurate than the 16 s sequencing we used to identify species.^50^, ^51^ But we have the advantage of having multi‐cohorts and low economic costs. Regarding alpha diversity, the previous meta‐analysis indicated that the α diversity is lower in healthy individuals, which is consistent with our results.^52^, ^53^ However, Jin et al. reported that the α diversity was lower in the LC population and decreased steadily at more advanced stages.^16^ Interestingly, similar trends were seen in the study by Greathouse et al. using lung tissue instead of BALF.^54^ Thus, α diversity may not be an appropriate indicator of lung health. Pulmonary disease alters the composition of LM, as evidenced by an increase in pathogenic bacteria such as *Escherichia coli* (*E. coli*), *Streptococcus pneumoniae*, and *Haemophilus influenza*.^55^ This phenomenon was also observed in our study. *E. coli* was selected as our biomarker. It is an opportunistic pathogen strongly associated with carcinogenesis (colorectal and bladder cancer, etc.).^56^, ^57^, ^58^ However, the mechanism by which it plays a role in lung carcinogenesis is still unclear, and only its potential relevance has been demonstrated.^59^ Some studies have confirmed that *E. coli* can promote the induction of IFN‐α, IFN‐β, and ISG in lung cells, eventually leading to lung injury.^60^, ^61^ *E. coli* was also detectable in individuals with COVID‐19, asthma, COPD, and other lung diseases.^62^, ^63^, ^64^ *E. coli*, as a typical parasitic bacterium of the gut, is frequently found in the lungs of LC patients. Its transmission routes, including the fecal‐oral or bloodstream transmission route, need to be explored in future studies.

In terms of LC diagnosis, in addition to the single microbiota study, the combination of multiple biomarkers also exhibits good performance for diagnosing LC. According to the results described by Lu et al., a model using a combination of micro biomarkers (AUC_gut_ + _sputum_ = 0.82) showed improved performance for patient stratification compared to a model using an individual dataset (AUC_gut_ = 0.76; AUC_sputum_ = 0.75).^20^ Notably, in studies of other cancer types, the use of a combination of different biomarkers (carcinoembryonic antigen, carbohydrate antigen, blood microorganisms, etc.) for the construction of classifiers has increased the predictive power compared to that observed with original single microbial data.^38^, ^65^, ^66^ Some studies also included clinical indicators such as age and BMI to enhance the accuracy of the diagnosis.^67^ Unfortunately, there is no dual‐model research on combining microbiota with blood in the LC field. Only a few correlation studies have been conducted. For instance, Chen et al. and Wang et al. used the Spearman rank correlation test to determine the association between different microorganisms and metabolites and the mechanism of potential bacterial flora involved in interventions in the metabolism and development of new blood biomarkers.^18^, ^19^ We shall attempt to meta‐analyze more types of biomarkers that help to improve our diagnostic performance in future studies.

Besides diagnosis, ML also has promising applications in the prognosis, classification, grading, and treatment optimization of cancer patients.^68^ For example, *Acidovorax* and *Veillonella* in the sputum can help diagnose squamous cell carcinoma with 80% sensitivity, while *Capnocytophaga* can be used to identify lung adenocarcinoma with 72% sensitivity.^22^ Regarding prognosis, both LM and GM significantly differ between groups exhibiting long and short progression‐free survival durations.^69^, ^70^ Based on the oral microbiota, the predictive potential could reach 0.89.^23^ In terms of the therapeutic effects of chemotherapy or immunotherapy, numerous studies have been carried out to screen response micro biomarkers and predict clinical side effects.^40^, ^71^, ^72^ Interestingly, microbiome‐based classifiers all use RF algorithms. It can be seen that the RF classifier has become the preferred choice for the study of this kind of problem.^73^ Notably, extensive literature reviews have shown that less attention has been focused on targeted therapy and radiotherapy, representing a significant gap in the research on LC‐related microbiota. Furthermore, ML techniques have been used to improve drug research, drug discovery, pharmacokinetic prediction, and drug treatment prediction.^74^, ^75^ For example, AI‐based drug–drug interaction prediction models have been constructed to facilitate the fundamental application of drug therapy and support clinical decisions.^76^ Hung TNK et al. extracted datasets from the DrugBank database as raw information. Then, ML algorithms (RF and XGBoost) were used to construct ML models for predicting the DDIs of the Osteoporosis‐Paget disease, which exhibited an average accuracy of nearly 74%.^77^ However, the use of AI for LC drug selection has not been developed effectively yet. In the future, it would still be necessary to study the optimal decision algorithms for selecting the best compounds and provide personalized drug therapy to LC patients.^78^ In addition to traditional ML algorithms, deep learning can help build deep networks continuously and learn and approximate real models.^79^ This particular ML technology would help us achieve powerful learning and diagnostic capabilities in the future. However, the generalizability of the data and the interpretability of the algorithm, the “black‐box problem,” and the problems of data access and medical ethics in the real‐world application are all challenges we face in the future.^80^

Despite our findings, there are some limitations associated with this study. First, we cannot obtain information on fungi and viruses using 16 s rRNA sequencing technology. Hence, the scope of research would be limited to bacteria. Moreover, this functional prediction and taxonomic resolution by 16 s rRNA data are not as good as that of WGS data. However, the cost of WGS for extensive screening needs to be considered. Second, our sample size and sampling area need to be expanded, and a larger sample size from multiple centers is required for modeling and validation. Finally, transcriptomics and metabolomics studies should also validate functional analysis results. Despite their limitations, the present results are of great significance for identifying LC biomarkers and shall contribute to the study of pathogenesis in the future. In particular, the discovery of gut biomarkers has been beneficial for the non‐invasive diagnosis of LC. Diagnostic accuracy can be improved in future studies by refining subtypes, determining LC stages, and considering more clinical indexes. Moreover, the combination of ML with real‐world data streams such as genomics, pathology, and electronic health records would help facilitate a powerful electronic synthesis that would be necessary for the further development of modern medicine.

## 5 CONCLUSION

In summary, we analyzed the composition of the gut and lung microbiome of LC patients via 16 s rRNA gene sequencing and constructed a diagnostic model in this study. The results show that LM has a higher diagnostic value than GM. However, GM is a promising candidate for developing non‐invasive diagnostics for a wide range of early screening‐related processes. Moreover, we also screened some micro biomarkers based on multi‐population samples that may be useful as entry points for targets that suppress LC carcinogenesis in the future. Our findings support the opinion that the LC population has a characteristic microbial composition and could provide some insight into the development of microbiology‐based early screening kits.

## AUTHOR CONTRIBUTIONS

**Wenjie Han:** Data curation (lead); investigation (lead); writing – original draft (lead). **Na Wang:** Conceptualization (supporting). **Mengzhen Han:** Data curation (equal). **Xiaolin Liu:** Visualization (equal). **Tao Sun:** Writing – review and editing (lead). **Junnan Xu:** Validation (lead).

## FUNDING INFORMATION

This work was supported by National Nature Science Foundation of China (82373113, XJ), Shenyang Breast Cancer Clinical Medical Research Center (2020‐48‐3‐1, ST), Liaoning Cancer Hospital Yangtse River Scholars Project (ST, XJ), LiaoNing Revitalization Talents Program (XLYC1907160, XJ), Beijing Medical Award Foundation (YXJL‐2020‐0941‐0752, ST), Wu Jieping Medical Foundation (320.6750.2020‐12‐21,320.6750.2020‐6‐30, ST) and the Fundamental Research Funds for the Central Universities (202229, ST; 202230, XJ).

## CONFLICT OF INTEREST STATEMENT

Author XL is employed by Liaoning Kanghui Biotechnology Co., Ltd. The remaining authors declare that the research was conducted in the absence of any commercial or financial relationships that could be construed as a potential conflict of interest.

## ETHICS STATEMENT

The study was approved by the Medical Ethics Committee of Liaoning Cancer Hospital, Shenyang, China (20220810YG).

## Supporting information


Supplementary Figure 1.
Supplementary Figure 2.
Supplementary Figure 3.
Supplementary Figure 4.
Supplementary Figure 5.
Supplementary Figure 6.
Supplementary Table 1.
Supplementary Table 2.
Supplementary Table 3.
Supplementary Table 4.
Supplementary Table 5.
Supplementary Table 6.
Supplementary Table 7.
Supplementary Table 8.

## Data Availability

Data sharing is not applicable to this article as no new data were created or analyzed in this study.

## References

[1] Thai AA , Solomon BJ , Sequist LV , Gainor JF , Heist RS . Lung cancer. Lancet. 2021;398(10299):535‐554. doi:10.1016/S0140-6736(21)00312-3 34273294

[2] Dong Q , Chen ES , Zhao C , Jin C . Host‐microbiome interaction in lung cancer. Front Immunol. 2021;12:679829. doi:10.3389/fimmu.2021.679829 34108973 PMC8183378

[3] Xu K , Zhang C , Du T , et al. Progress of exosomes in the diagnosis and treatment of lung cancer. Biomed Pharmacother. 2021;134:111111. doi:10.1016/j.biopha.2020.111111 33352449

[4] Oudkerk M , Liu S , Heuvelmans MA , Walter JE , Field JK . Lung cancer Ldct screening and mortality reduction ‐ evidence, pitfalls and future perspectives. Nat Rev Clin Oncol. 2021;18(3):135‐151. doi:10.1038/s41571-020-00432-6 33046839

[5] Nooreldeen R , Bach H . Current and future development in lung cancer diagnosis. Int J Mol Sci. 2021;22(16):8661. doi:10.3390/ijms22168661 34445366 PMC8395394

[6] Hamet P , Tremblay J . Artificial intelligence in medicine. Metabolism. 2017;69S:S36‐S40. doi:10.1016/j.metabol.2017.01.011 28126242

[7] Dilsizian SE , Siegel EL . Artificial intelligence in medicine and cardiac imaging: harnessing big data and advanced computing to provide personalized medical diagnosis and treatment. Curr Cardiol Rep. 2014;16(1):441. doi:10.1007/s11886-013-0441-8 24338557

[8] Menden MP , Iorio F , Garnett M , et al. Machine learning prediction of cancer cell sensitivity to drugs based on genomic and chemical properties. PloS One. 2013;8(4):e61318. doi:10.1371/journal.pone.0061318 23646105 PMC3640019

[9] Cruz JA , Wishart DS . Applications of machine learning in cancer prediction and prognosis. Cancer Inform. 2007;2:59‐77. 19458758 PMC2675494

[10] Chen Z‐H , Lin L , Wu C‐F , Li C‐F , Xu R‐H , Sun Y . Artificial intelligence for assisting cancer diagnosis and treatment in the era of precision medicine. Cancer Commun (Lond). 2021;41(11):1100‐1115. doi:10.1002/cac2.12215 34613667 PMC8626610

[11] Garrett WS . Cancer and the microbiota. Science (New York, NY). 2015;348(6230):80‐86. doi:10.1126/science.aaa4972 PMC5535753 25838377

[12] Budden KF , Gellatly SL , Wood DLA , et al. Emerging pathogenic links between microbiota and the gut‐lung Axis. Nat Rev Microbiol. 2017;15(1):55‐63. doi:10.1038/nrmicro.2016.142 27694885

[13] Marshall EA , Filho FSL , Sin DD , Lam S , Leung JM , Lam WL . Distinct bronchial microbiome precedes clinical diagnosis of lung cancer. Mol Cancer. 2022;21(1):68. doi:10.1186/s12943-022-01544-6 35255902 PMC8900294

[14] Guo Y , Yuan W , Lyu N , et al. Association studies on gut and lung microbiomes in patients with lung adenocarcinoma. Microorganisms. 2023;11(3):546. doi:10.3390/microorganisms11030546 36985120 PMC10059697

[15] Zheng X , Sun X , Liu Q , Huang Y , Yuan Y . The composition alteration of respiratory microbiota in lung cancer. Cancer Invest. 2020;38(3):158‐168. doi:10.1080/07357907.2020.1732405 32073913

[16] Jin J , Gan Y , Liu H , et al. Diminishing microbiome richness and distinction in the lower respiratory tract of lung cancer patients: a multiple comparative study design with independent validation. Lung Cancer. 2019;136:129‐135. doi:10.1016/j.lungcan.2019.08.022 31494531

[17] Liu F , Li J , Guan Y , et al. Dysbiosis of the gut microbiome is associated with tumor biomarkers in lung cancer. Int J Biol Sci. 2019;15(11):2381‐2392. doi:10.7150/ijbs.35980 31595156 PMC6775324

[18] Chen S , Gui R , Zhou X‐H , et al. Combined microbiome and metabolome analysis reveals a novel interplay between intestinal Flora and Serum metabolites in lung cancer. Front Cell Infect Microbiol. 2022;12:885093. doi:10.3389/fcimb.2022.885093 35586253 PMC9108287

[19] Wang S , Chen H , Yang H , et al. Gut microbiome was highly related to the regulation of metabolism in lung adenocarcinoma patients. Front Oncol. 2022;12:790467. doi:10.3389/fonc.2022.790467 35592677 PMC9113755

[20] Lu H , Gao NL , Tong F , et al. Alterations of the human lung and gut microbiomes in non‐small cell lung carcinomas and distant metastasis. Microbiol Spectr. 2021;9(3):e0080221. doi:10.1128/Spectrum.00802-21 34787462 PMC8597645

[21] An R , He DD , Zhao F , Wang LQ , Wang XJ . Analysis of gut microbiome in patients with lung adenocarcinoma and lung squamous cell carcinoma. Zhonghua Yu Fang Yi Xue Za Zhi. 2021;55(5):679‐684. doi:10.3760/cma.j.cn112150-20200826-01155 34034411

[22] Leng Q , Holden VK , Deepak J , Todd NW , Jiang F . Microbiota biomarkers for lung cancer. Diagnostics (Basel). 2021;11:3. doi:10.3390/diagnostics11030407 PMC7997424 33673596

[23] Lim MY , Hong S , Hwang KH , Lim EJ , Han J‐Y , Nam Y‐D . Diagnostic and prognostic potential of the Oral and gut microbiome for lung adenocarcinoma. Clin Transl Med. 2021;11(9):e508. doi:10.1002/ctm2.508 34586729 PMC8473640

[24] Magoč T , Salzberg SL . Flash: fast length adjustment of short reads to improve genome assemblies. Bioinformatics (Oxford, England). 2011;27(21):2957‐2963. doi:10.1093/bioinformatics/btr507 21903629 PMC3198573

[25] Cock PJA , Fields CJ , Goto N , Heuer ML , Rice PM . The sanger Fastq file format for sequences with quality scores, and the Solexa/Illumina Fastq variants. Nucleic Acids Res. 2010;38(6):1767‐1771. doi:10.1093/nar/gkp1137 20015970 PMC2847217

[26] Wirbel J , Pyl PT , Kartal E , et al. Meta‐analysis of fecal metagenomes reveals global microbial signatures that are specific for colorectal cancer. Nat Med. 2019;25(4):679‐689. doi:10.1038/s41591-019-0406-6 30936547 PMC7984229

[27] Su Q , Liu Q , Lau RI , et al. Faecal microbiome‐based machine learning for multi‐class disease diagnosis. Nat Commun. 2022;13(1):6818. doi:10.1038/s41467-022-34405-3 36357393 PMC9649010

[28] Lin Y , Lau HC‐H , Liu Y , et al. Altered Mycobiota signatures and enriched pathogenic aspergillus Rambellii are associated with colorectal cancer based on multi‐cohort fecal metagenomic analyses. Gastroenterology. 2022;163(4):908‐921. doi:10.1053/j.gastro.2022.06.038 35724733

[29] Gibbons SM , Duvallet C , Alm EJ . Correcting for batch effects in case‐control microbiome studies. PLoS Comput Biol. 2018;14(4):e1006102. doi:10.1371/journal.pcbi.1006102 29684016 PMC5940237

[30] Sivaprakasam S , Prasad PD , Singh N . Benefits of short‐chain fatty acids and their receptors in inflammation and carcinogenesis. Pharmacol Ther. 2016;164:144‐151. doi:10.1016/j.pharmthera.2016.04.007 27113407 PMC4942363

[31] Bultman SJ . The microbiome and its potential as a cancer preventive intervention. Semin Oncol. 2016;43(1):97‐106. doi:10.1053/j.seminoncol.2015.09.001 26970128 PMC4789109

[32] Li M , Liu J , Zhu J , et al. Performance of gut microbiome as an independent diagnostic tool for 20 diseases: cross‐cohort validation of machine‐learning classifiers. Gut Microbes. 2023;15(1):2205386. doi:10.1080/19490976.2023.2205386 37140125 PMC10161951

[33] Richardson DB , Cole SR , Ross RK , Poole C , Chu H , Keil AP . Meta‐analysis and sparse‐data bias. Am J Epidemiol. 2021;190(2):336‐340. doi:10.1093/aje/kwaa205 32975277

[34] Hameed MAB , Alamgir Z . Improving mortality prediction in acute pancreatitis by machine learning and data augmentation. Comput Biol Med. 2022;150:106077. doi:10.1016/j.compbiomed.2022.106077 36137318

[35] Liu N‐N , Jiao N , Tan J‐C , et al. Multi‐kingdom microbiota analyses identify bacterial‐fungal interactions and biomarkers of colorectal cancer across cohorts. Nat Microbiol. 2022;7(2):238‐250. doi:10.1038/s41564-021-01030-7 35087227 PMC8813618

[36] Leibovitzh H , Lee S‐H , Xue M , et al. Altered gut microbiome composition and function are associated with gut barrier dysfunction in healthy relatives of patients with Crohn's disease. Gastroenterology. 2022;163(5):1364‐1376.e10. doi:10.1053/j.gastro.2022.07.004 35850197

[37] Leung H , Long X , Ni Y , et al. Risk assessment with gut microbiome and metabolite markers in Nafld development. Sci Transl Med. 2022;14(648):eabk0855. doi:10.1126/scitranslmed.abk0855 35675435 PMC9746350

[38] Kartal E , Schmidt TSB , Molina‐Montes E , et al. A Faecal microbiota signature with high specificity for pancreatic cancer. Gut. 2022;71(7):1359‐1372. doi:10.1136/gutjnl-2021-324755 35260444 PMC9185815

[39] Zhuang H , Cheng L , Wang Y , et al. Dysbiosis of the gut microbiome in lung cancer. Front Cell Infect Microbiol. 2019;9:112. doi:10.3389/fcimb.2019.00112 31065547 PMC6489541

[40] Zhao Z , Fei K , Bai H , Wang Z , Duan J , Wang J . Metagenome association study of the gut microbiome revealed biomarkers linked to chemotherapy outcomes in locally advanced and advanced lung cancer. Thorac Cancer. 2021;12(1):66‐78. doi:10.1111/1759-7714.13711 33111503 PMC7779204

[41] Fluckiger A , Daillère R , Sassi M , et al. Cross‐reactivity between tumor Mhc class I‐restricted antigens and an enterococcal bacteriophage. Science. 2020;369(6506):936‐942. doi:10.1126/science.aax0701 32820119

[42] Suprewicz Ł , Tokajuk G , Cieśluk M , et al. Bacteria residing at root canals can induce cell proliferation and Alter the mechanical properties of gingival and cancer cells. Int J Mol Sci. 2020;21(21):7914. doi:10.3390/ijms21217914 33114460 PMC7672538

[43] Liu W‐B , Han F , Huang Y‐S , et al. Tmem196 hypermethylation as a novel diagnostic and prognostic biomarker for lung cancer. Mol Carcinog. 2019;58(4):474‐487. doi:10.1002/mc.22942 30536447

[44] Wang H , Xu J , Ding L . Microrna‐21 was a promising biomarker for lung carcinoma diagnosis: An update meta‐analysis. Thorac Cancer. 2022;13(3):316‐321. doi:10.1111/1759-7714.14242 34837469 PMC8807252

[45] Yan X , Yang M , Liu J , et al. Discovery and validation of potential bacterial biomarkers for lung cancer. Am J Cancer Res. 2015;5(10):3111‐3122. 26693063 PMC4656734

[46] Peres RL , Palaci M , Loureiro RB , et al. Evaluation of oral antiseptic rinsing before sputum collection to reduce contamination of mycobacterial cultures. J Clin Microbiol. 2011;49(8):3058‐3060. doi:10.1128/JCM.00541-11 21677070 PMC3147721

[47] Huang D , Su X , Yuan M , et al. The characterization of lung microbiome in lung cancer patients with different clinicopathology. Am J Cancer Res. 2019;9(9):2047‐2063. 31598405 PMC6780665

[48] Bello S , Vengoechea JJ , Ponce‐Alonso M , et al. Core microbiota in central lung cancer with streptococcal enrichment as a possible diagnostic marker. Arch Bronconeumol. 2021;57(11):681‐689. doi:10.1016/j.arbr.2020.05.017 35699005

[49] Herth FJ , Mayer M , Thiboutot J , et al. Safety and performance of transbronchial cryobiopsy for parenchymal lung lesions. Chest. 2021;160(4):1512‐1519. doi:10.1016/j.chest.2021.04.063 33971147

[50] Ranjan R , Rani A , Metwally A , McGee HS , Perkins DL . Analysis of the microbiome: advantages of whole genome shotgun versus 16s amplicon sequencing. Biochem Biophys Res Commun. 2016;469(4):967‐977. doi:10.1016/j.bbrc.2015.12.083 26718401 PMC4830092

[51] Ma J , Prince A , Aagaard KM . Use of whole genome shotgun metagenomics: a practical guide for the microbiome‐minded physician scientist. Semin Reprod Med. 2014;32(1):5‐13. doi:10.1055/s-0033-1361817 24390915

[52] Zimmermann A , Knecht H , Häsler R , et al. Atopobium and fusobacterium as novel candidates for sarcoidosis‐associated microbiota. Eur Respir J. 2017;50(6):1600746. doi:10.1183/13993003.00746-2016 29242257

[53] Lee SH , Sung JY , Yong D , et al. Characterization of microbiome in bronchoalveolar lavage fluid of patients with lung cancer comparing with benign mass like lesions. Lung Cancer. 2016;102:89‐95. doi:10.1016/j.lungcan.2016.10.016 27987594

[54] Greathouse KL , White JR , Vargas AJ , et al. Interaction between the microbiome and Tp53 in human lung cancer. Genome Biol. 2018;19(1):123. doi:10.1186/s13059-018-1501-6 30143034 PMC6109311

[55] Berghmans T , Sculier J‐P , Klastersky J . A prospective study of infections in lung cancer patients admitted to the hospital. Chest. 2003;124(1):114‐120. 12853512 10.1378/chest.124.1.114

[56] Denamur E , Clermont O , Bonacorsi S , Gordon D . The population genetics of pathogenic Escherichia Coli. Nat Rev Microbiol. 2021;19(1):37‐54. doi:10.1038/s41579-020-0416-x 32826992

[57] Nouri R , Hasani A , Shirazi KM , et al. Escherichia Coli and colorectal cancer: unfolding the enigmatic relationship. Curr Pharm Biotechnol. 2022;23(10):1257‐1268. doi:10.2174/1389201022666210910094827 34514986

[58] Abd‐El‐Raouf R , Ouf SA , Gabr MM , Zakaria MM , El‐Yasergy KF , Ali‐El‐Dein B . Escherichia Coli Foster bladder cancer cell line progression via epithelial mesenchymal transition, stemness and metabolic reprogramming. Sci Rep. 2020;10(1):18024. doi:10.1038/s41598-020-74390-5 33093503 PMC7581527

[59] Wong LM , Shende N , Li WT , et al. Comparative analysis of age‐ and gender‐associated microbiome in lung adenocarcinoma and lung squamous cell carcinoma. Cancers (Basel). 2020;12(6):1447. doi:10.3390/cancers12061447 32498338 PMC7352186

[60] Jin Y , Jia Z , Cai Q , Sun Y , Liu Z . Escherichia coli infection activates the production of Ifn‐Α and Ifn‐Β via the Jak1/Stat1/2 signaling pathway in lung cells. Amino Acids. 2021;53(10):1609‐1622. doi:10.1007/s00726-021-03077-6 34524541 PMC8441250

[61] Liu QX , Zhou Y , Li XM , et al. Ammonia induce lung tissue injury in broilers by activating Nlrp3 inflammasome via Escherichia/shigella. Poult Sci. 2020;99(7):3402‐3410. doi:10.1016/j.psj.2020.03.019 32616234 PMC7597683

[62] Baskaran V , Lawrence H , Lansbury LE , et al. Co‐infection in critically ill patients with Covid‐19: An observational cohort study from England. J Med Microbiol. 2021;70(4):001350. doi:10.1099/jmm.0.001350 33861190 PMC8289210

[63] Castro‐Nallar E , Shen Y , Freishtat RJ , et al. Integrating microbial and host transcriptomics to characterize asthma‐associated microbial communities. BMC Med Genomics. 2015;8:50. doi:10.1186/s12920-015-0121-1 26277095 PMC4537781

[64] Ye F , He L‐X , Cai B‐Q , et al. Spectrum and antimicrobial resistance of common pathogenic bacteria isolated from patients with acute exacerbation of chronic obstructive pulmonary disease in mainland of China. Chin Med J (Engl). 2013;126(12):2207‐2214. 23786927

[65] Zhou C‐B , Pan S‐Y , Jin P , et al. Fecal signatures of streptococcus Anginosus and streptococcus constellatus for non‐invasive screening and early warning of gastric cancer. Gastroenterology. 2022;162(7):1933‐1947.e18. doi:10.1053/j.gastro.2022.02.015 35167866

[66] Poore GD , Kopylova E , Zhu Q , et al. Microbiome analyses of blood and tissues suggest cancer diagnostic approach. Nature. 2020;579(7800):567‐574. doi:10.1038/s41586-020-2095-1 32214244 PMC7500457

[67] Wu Y , Jiao N , Zhu R , et al. Identification of microbial markers across populations in early detection of colorectal cancer. Nat Commun. 2021;12(1):3063. doi:10.1038/s41467-021-23265-y 34031391 PMC8144394

[68] Elemento O , Leslie C , Lundin J , Tourassi G . Artificial intelligence in cancer research diagnosis and therapy. Nat Rev Cancer. 2021;21(12):747‐752. doi:10.1038/s41568-021-00399-1 34535775

[69] Gomes S , Cavadas B , Ferreira JC , et al. Profiling of lung microbiota discloses differences in adenocarcinoma and squamous cell carcinoma. Sci Rep. 2019;9(1):12838. doi:10.1038/s41598-019-49195-w 31492894 PMC6731246

[70] Xi Y , Liu F , Qiu B , et al. Analysis of gut microbiota signature and microbe‐disease progression associations in locally advanced non‐small cell lung cancer patients treated with concurrent chemoradiotherapy. Front Cell Infect Microbiol. 2022;12:892401. doi:10.3389/fcimb.2022.892401 35719339 PMC9200620

[71] Zhang M , Liu D , Zhou H , et al. Intestinal Flora characteristics of advanced non‐small cell lung cancer in China and their role in chemotherapy based on metagenomics: a prospective exploratory cohort study. Thorac Cancer. 2021;12(24):3293‐3303. doi:10.1111/1759-7714.14199 34693651 PMC8671906

[72] Liu T , Xiong Q , Li L , Hu Y . Intestinal microbiota predicts lung cancer patients at risk of immune‐related diarrhea. Immunotherapy. 2019;11(5):385‐396. doi:10.2217/imt-2018-0144 30693820

[73] Uddin S , Khan A , Hossain ME , Moni MA . Comparing different supervised machine learning algorithms for disease prediction. BMC Med Inform Decis Mak. 2019;19(1):281. doi:10.1186/s12911-019-1004-8 31864346 PMC6925840

[74] Kirchmair J , Göller AH , Lang D , et al. Predicting drug metabolism: experiment and/or computation? Nat Rev Drug Discov. 2015;14(6):387‐404. doi:10.1038/nrd4581 25907346

[75] You Y , Lai X , Pan Y , et al. Artificial intelligence in cancer target identification and drug discovery. Signal Transduct Target Ther. 2022;7(1):156. doi:10.1038/s41392-022-00994-0 35538061 PMC9090746

[76] Vo TH , Nguyen NTK , Kha QH , Le NQK . On the road to explainable ai in drug‐drug interactions prediction: a systematic review. Comput Struct Biotechnol J. 2022;20:2112‐2123. doi:10.1016/j.csbj.2022.04.021 35832629 PMC9092071

[77] Hung TNK , Le NQK , Le NH , et al. An ai‐based prediction model for drug‐drug interactions in osteoporosis and Paget's diseases from smiles. Mol Inform. 2022;41(6):e2100264. doi:10.1002/minf.202100264 34989149

[78] Pei Q , Luo Y , Chen Y , Li J , Xie D , Ye T . Artificial intelligence in clinical applications for lung cancer: diagnosis, treatment and prognosis. Clin Chem Lab Med. 2022;60(12):1974‐1983. doi:10.1515/cclm-2022-0291 35771735

[79] Huang S , Yang J , Shen N , Xu Q , Zhao Q . Artificial intelligence in lung cancer diagnosis and prognosis: current application and future perspective. Semin Cancer Biol. 2023;89:30‐37. doi:10.1016/j.semcancer.2023.01.006 36682439

[80] Kundu S . Ai in medicine must Be explainable. Nat Med. 2021;27(8):1328. doi:10.1038/s41591-021-01461-z 34326551

