# Supplementary material for: Identification of microbial markers associated with lung cancer based on multi‐cohort 16 s rRNA analyses: A systematic review and meta‐analysis
Source: Cancer Med. 2023 Sep 7;12(18):19301–19. doi: 10.1002/cam4.6503 (PMC10557844; doi:10.1002/cam4.6503)
Supplement: Supplementary file 1 — Supplementary Figure 1. Supplementary Figure 2. Supplementary Figure 3. Supplementary Figure 4. Supplementary Figure 5. Supplementary Figure 6. Supplementary Table 1. Supplementary Table 2. Supplementary Table 3. Supplementary Table 4. Supplementary Table 5. Supplementary Table 6. Supplementary Table 7. Supplementary Table 8. [file CAM4-12-19301-s001.docx]

**Identification of microbial markers associated with lung cancer based on multicohort 16s rRNA analyses: A systematic review and meta-analysis**

Wenjie Han ^1,2^, Mengzhen Han^1,2^, Yiwen Wang ^1,2^, Na Wang ^1,2^, Xiaolin Liu^3^, Jianying Dai^4^, Yuesheng Dong^4^, Tao Sun^1,5,6^ and Junnan Xu^1,2,6*^

**Content**

[1 Electronic search strategy in PubMed and Embase databases 2](#_Toc136286134)

[2 Development of machine learning models and tuning hyperparameter 3](#_Toc136286135)

[2.1 Development of Machine Learning Models 3](#_Toc136286136)

[2.2 The selection strategy of optimal hyperparameters for the RF model 4](#_Toc136286137)

[3 Supplementary Figures 6](#_Toc136286138)

[4 Supplementary Tables 11](#_Toc136286139)

#

# Electronic search strategy in PubMed and Embase databases

**MEDLINE (PUBMED)**

1. (gut OR gastrointestinal OR intestinal OR fecal OR fecal OR stool OR respiratory OR lung OR bronchoalveolar lavage fluid OR bronchial brushes) [tiab]
2. (microbiome OR microbiota OR ecosystem OR bacteria OR flora OR microflora OR dysbiosis) [tiab]
3. ("lung cancer" OR 'lung neoplasm" OR "pulmonary neoplasm" OR "pulmonary cancer' OR "lung adenocarcinoma" OR "Squamous cell lung carcinoma") AND（cancer or tumor or carcinoma）[tiab]
4. (Metagenomic OR 16s rRNA OR transcriptome OR meta-analysis) [tiab]
5. 1 AND 2 AND 3 AND 4

**EMBASE**

1. (gut OR gastrointestinal OR intestinal OR fecal OR fecal OR stool OR respiratory OR lung OR bronchoalveolar lavage fluid OR bronchial brushes) [tiab]
2. (microbiome OR microbiota OR ecosystem OR bacteria OR flora OR microflora OR dysbiosis) [tiab]
3. ("lung cancer" OR 'lung neoplasm" OR "pulmonary neoplasm" OR "pulmonary cancer' OR "lung adenocarcinoma" OR "Squamous cell lung carcinoma") AND（cancer or tumor or carcinoma）[tiab]
4. (Metagenomic OR 16s rRNA OR transcriptome OR meta-analysis) [tiab]
5. 1 AND 2 AND 3 AND 4

# Development of machine learning models and tuning hyperparameter

## Development of Machine Learning Models

We trained three common machine learning multi-class classifiers (K-nearest neighbors (KNN), support vector machine (SVM), and random forest (RF)) based on the four cohorts we collected. The 70% samples are the training set, and the 30% are the test set. All of these models had an AUROC of 0.780-0.807 ([Figure 2](https://www.ncbi.nlm.nih.gov/pmc/articles/PMC9273930/table/T2/).1). According to 10-fold cross-validation calculations, the RF model's performance in the test set greatly beat that of all other models and was comparable to that of the training set, indicating good integrity for this classifier. RF showed better specificity, sensitivity, accuracy, and kappa efficiency than the other models ([Table 2](https://www.ncbi.nlm.nih.gov/pmc/articles/PMC9273930/table/T2/).1). Therefore, subsequent studies were conducted using the RF multi-class model.


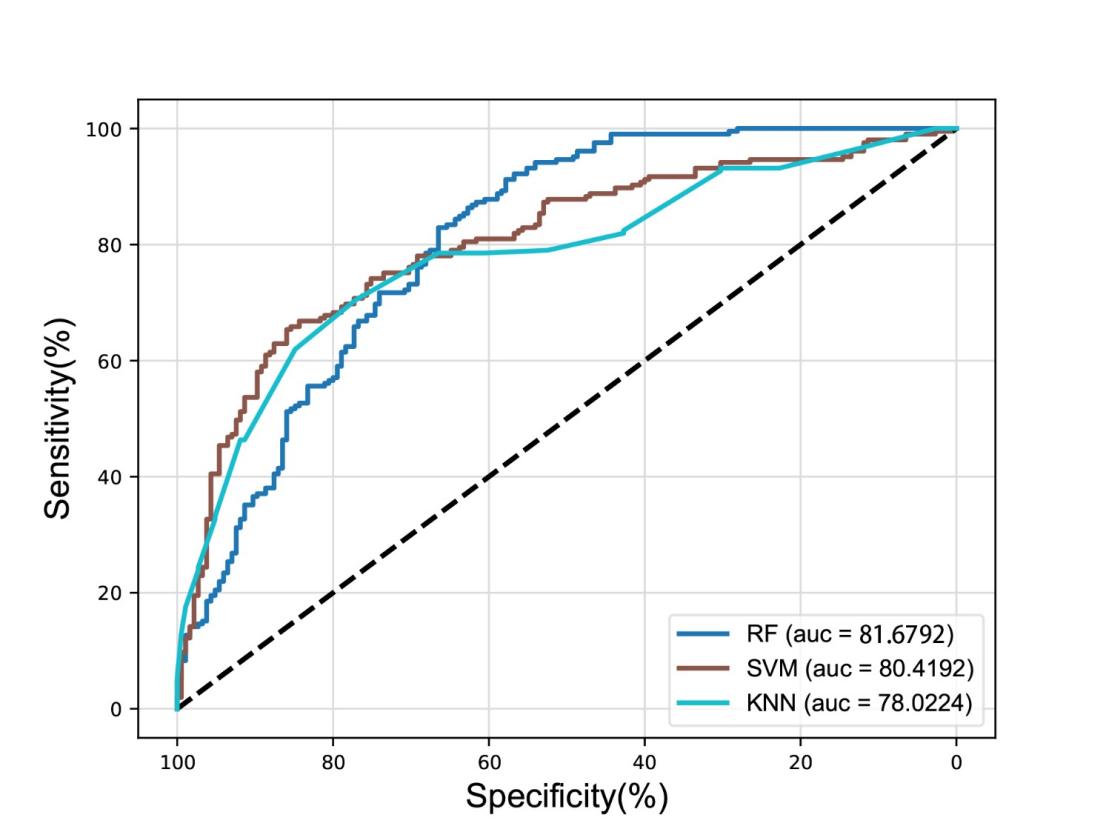


**Figure 2.1**. The support vector machine (SVM), random forest (RF), and K-nearest neighbors (KNN) algorithms were used to train three machine learning models. The area under the receiver operating characteristic curve (AUROC) of these models. The AUC curves of the three models are shown in the figure.

**Table 2.1** Performance metric details of three machine learning algorithms in testing sets

| Model | Specificity | Sensitivity | Accuracy | Kappa coefficient |
| --- | --- | --- | --- | --- |
| RF | 0.637 | 0.961 | 0.805 | 0.605 |
| KNN | 0.782 | 0.672 | 0.724 | 0.450 |
| SVM | 0.690 | 0.797 | 0.744 | 0.488 |

K-nearest neighbors (KNN), support vector machine (SVM), and random forest (RF)

## The selection strategy of optimal hyperparameters for the RF model

The gut model is taken as an example to explain the process of hyper-parameter tuning. The R (3.6.1) environment was used to implement the tuning hyperparameters for this study. Since the model is a classification model, "mtry" is set to the square root of the data set's variable count. And in terms of "ntree", our strategy is preserving the default value of "mtry" and testing multiple values. The best value of "ntree" is selected by evaluating the classification error. As can be seen from the figure, when the value of "ntree" is equal to 800, the model reaches the minimum classification error. We finally determined that the hyperparameter "ntree" was 800, and other parameters used default parameters.


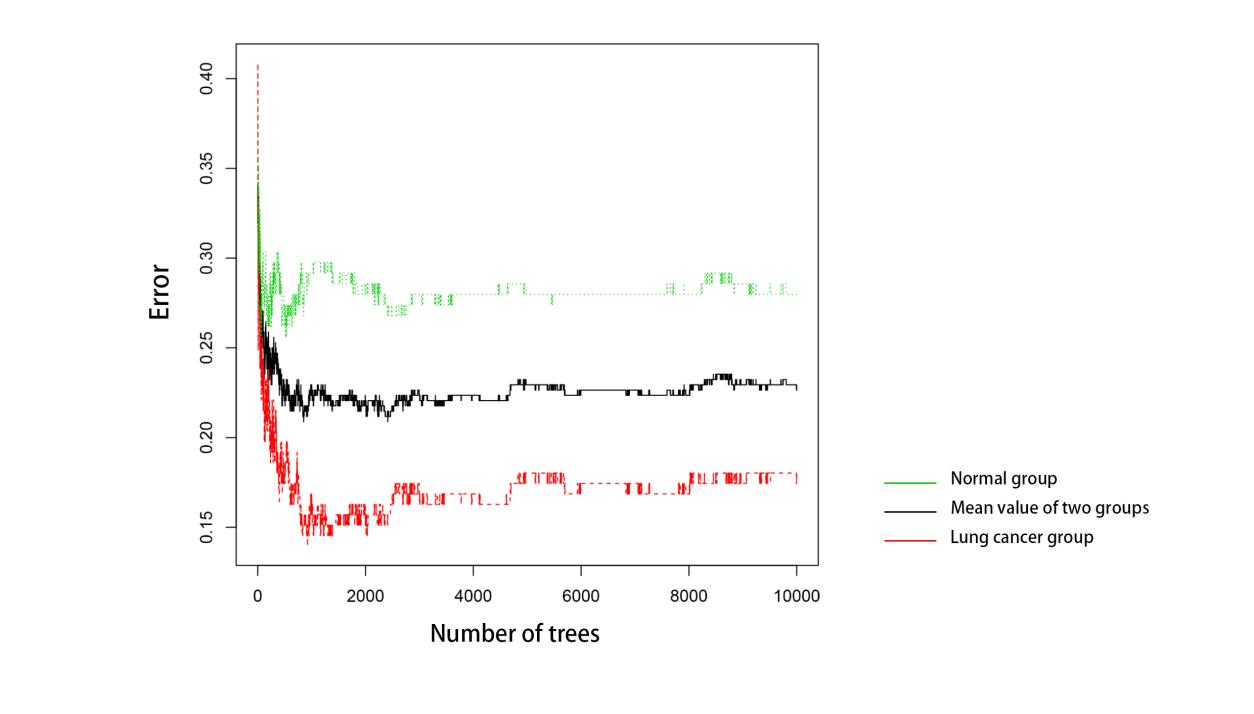


**Figure 2.2**. The quantitative relationship between "ntree" and classification error rate.

In terms of selecting the optimal feature variables, R language package randomForest() will evaluate the importance of the variables in the training model stage, and the results will output two indexes: "Mean Decrease Accuracy" and "Mean Decrease Gini." We have chosen the more commonly used "Mean Decrease Accuracy" for importance ranking here. Then, the relationship between the model error and the number of feature variables used for fitting was analyzed with 10-fold cross-validation. The result indicates that the error is minimum when the number of variables is near 35. Therefore, we selected a different number of variables, around 35, for modeling. As shown in **Figure 2.3**, when the number of variables is 36, the AUC value reaches the highest. Therefore, we finally selected top36 genera as our feature variables to construct the RF model.


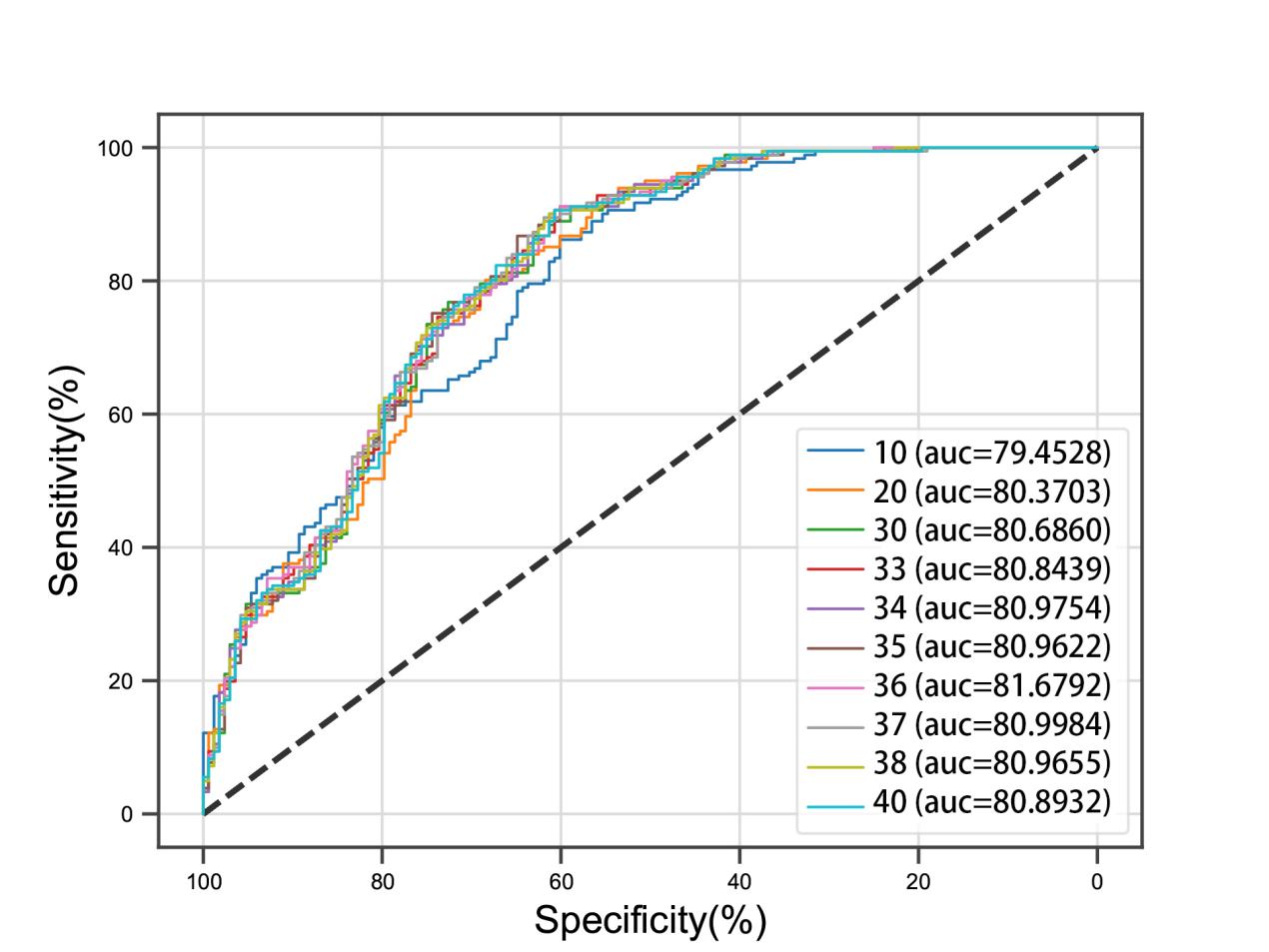


**Figure 2.3.** The AUC of the random forest models constructed with different numbers of feature variables.

# Supplementary Figures


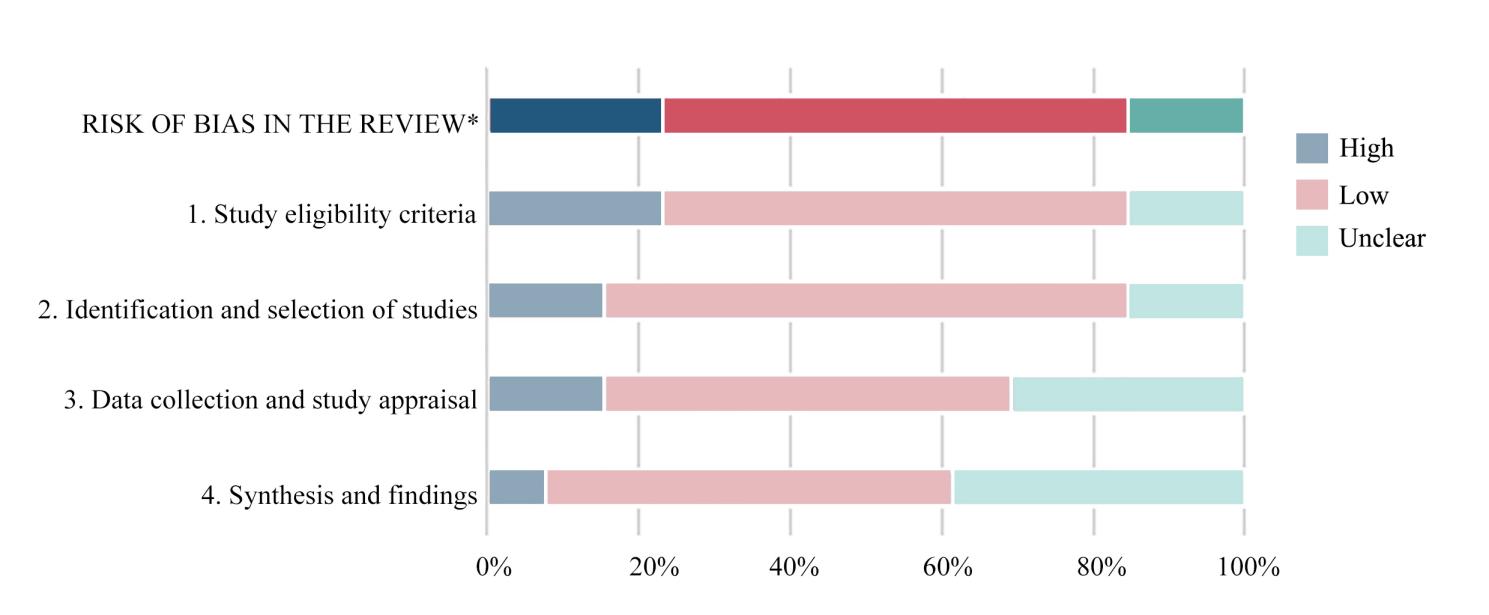


**Supplementary Figure 1.** Quality assessment of the included systematic reviews using the ROBIS tool.


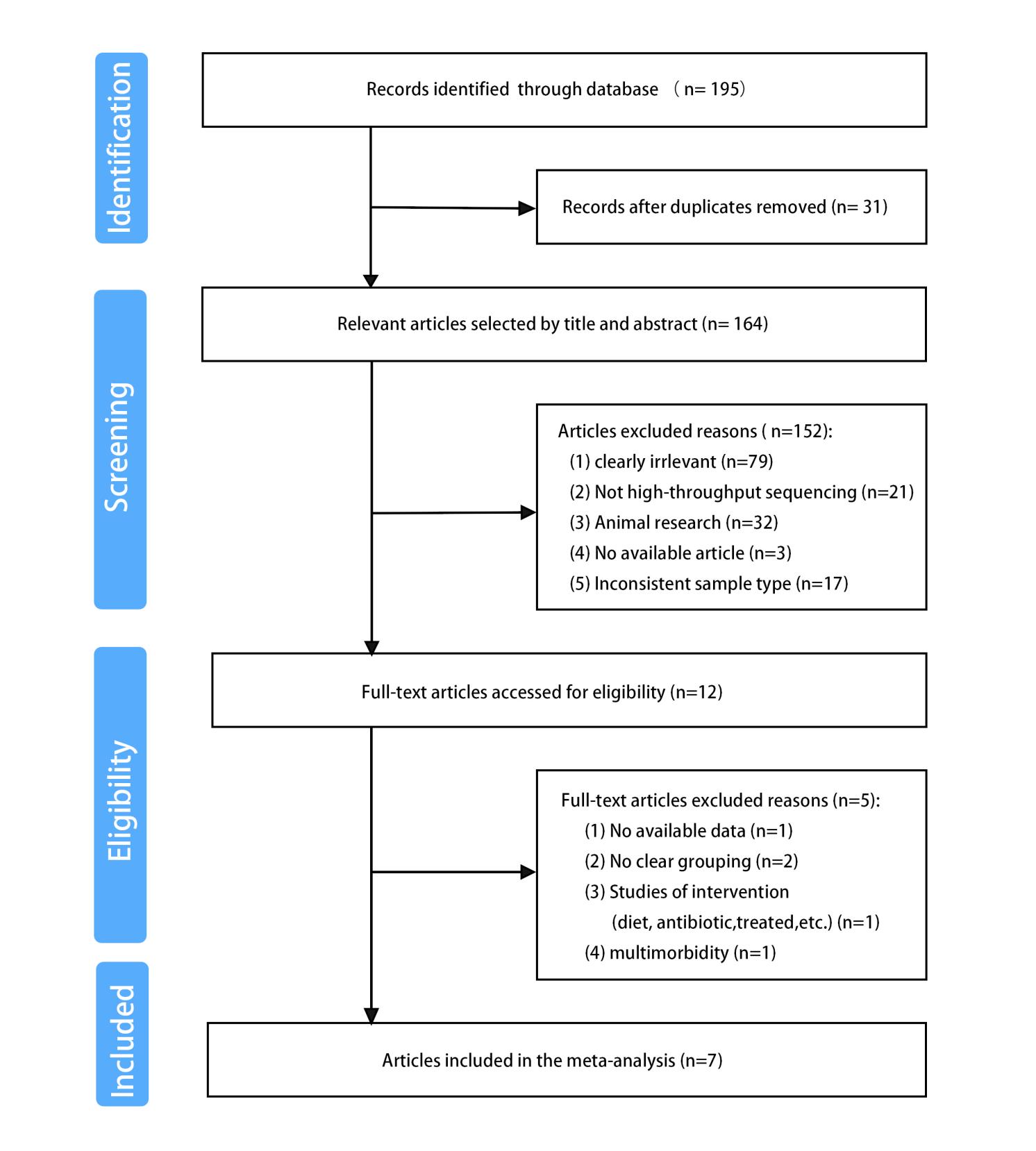


**Supplementary Figure 2.** PRISMA flowcharts for the selection process of the included studies. PRISMA, Preferred Reporting Items for Systematic Reviews and Meta-Analyses.


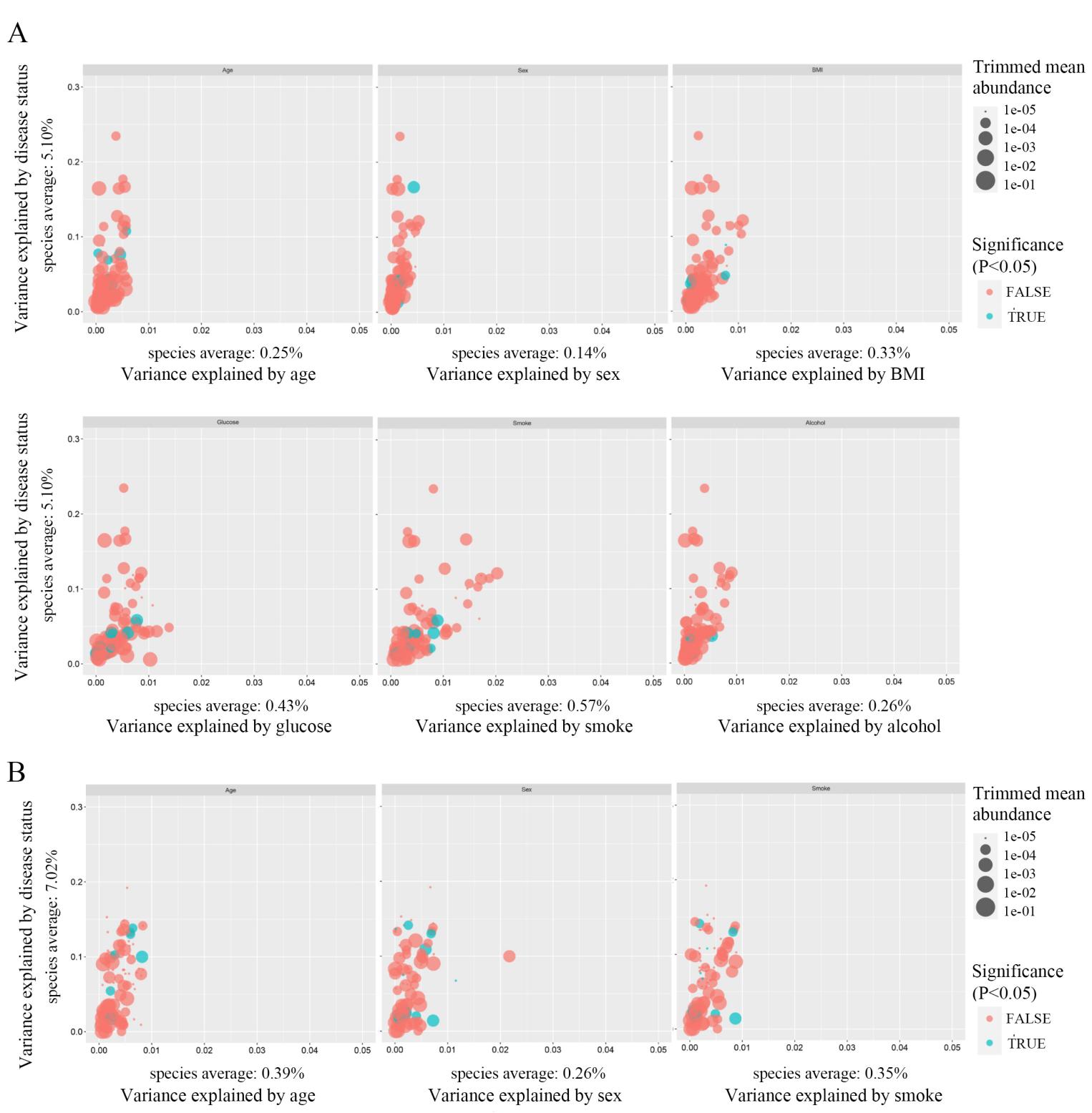


**Supplementary Figure 3.** Putative confounding factors and disease status explain the total variance. (A) For the gut study, the clinical indicators of age, sex, BMI, glucose, smoking history, and alcohol history be taken into account. (B) For lung study, the clinical indicators of age, sex, and smoke history be included in the examination. The significantly differential OTUs are colored in blue, and P values were from the two-way ANOVA test (see Methods 2.5.1). BMI, body mass index; OTUs, operational taxonomic units; ANOVA, analysis of variance.


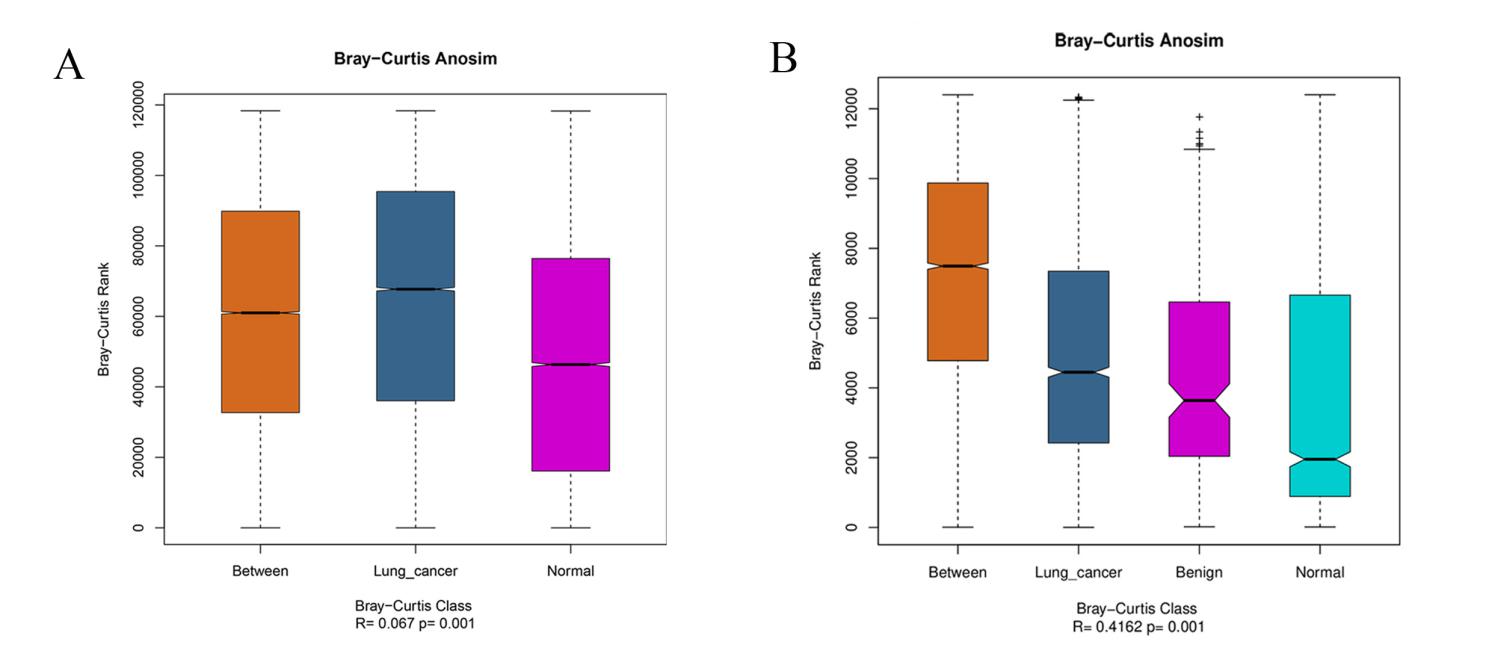


**Supplementary Figure 4.** The significant difference between the microbiota in the samples of the groups according to the analysis of similarities (ANOSIM). (A) The ANOSIM based on Bray Curtis demonstrated differences in gut microbiota composition between the LC and Normal groups (P = 0.001). (B) In the lung microbiota, the ANOSIM showed significant differences between the LC, Benign, and Normal groups (P = 0.001). ANOSIM, analysis of similarities.


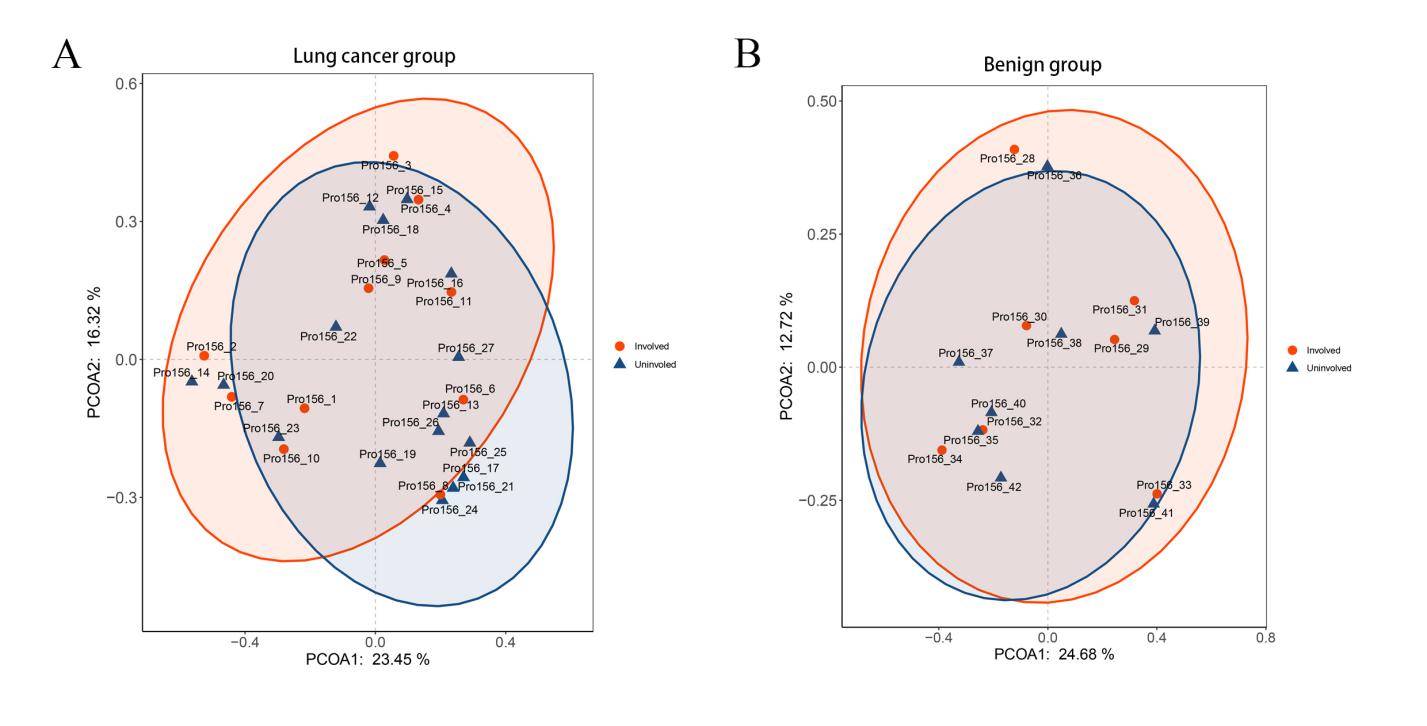


**Supplementary Figure 5.** Differences in bacterial community structure between involved and uninvolved airways. The analysis results for patients with lung cancer are shown on the left, and the results for patients with benign lung disease are on the right. Microbiota differences between involved and uninvolved airways were explored using PCoA analysis based on the Bray-Curtis Dissimilarity index (p>0.001). PCoA, principal coordinate analysis.


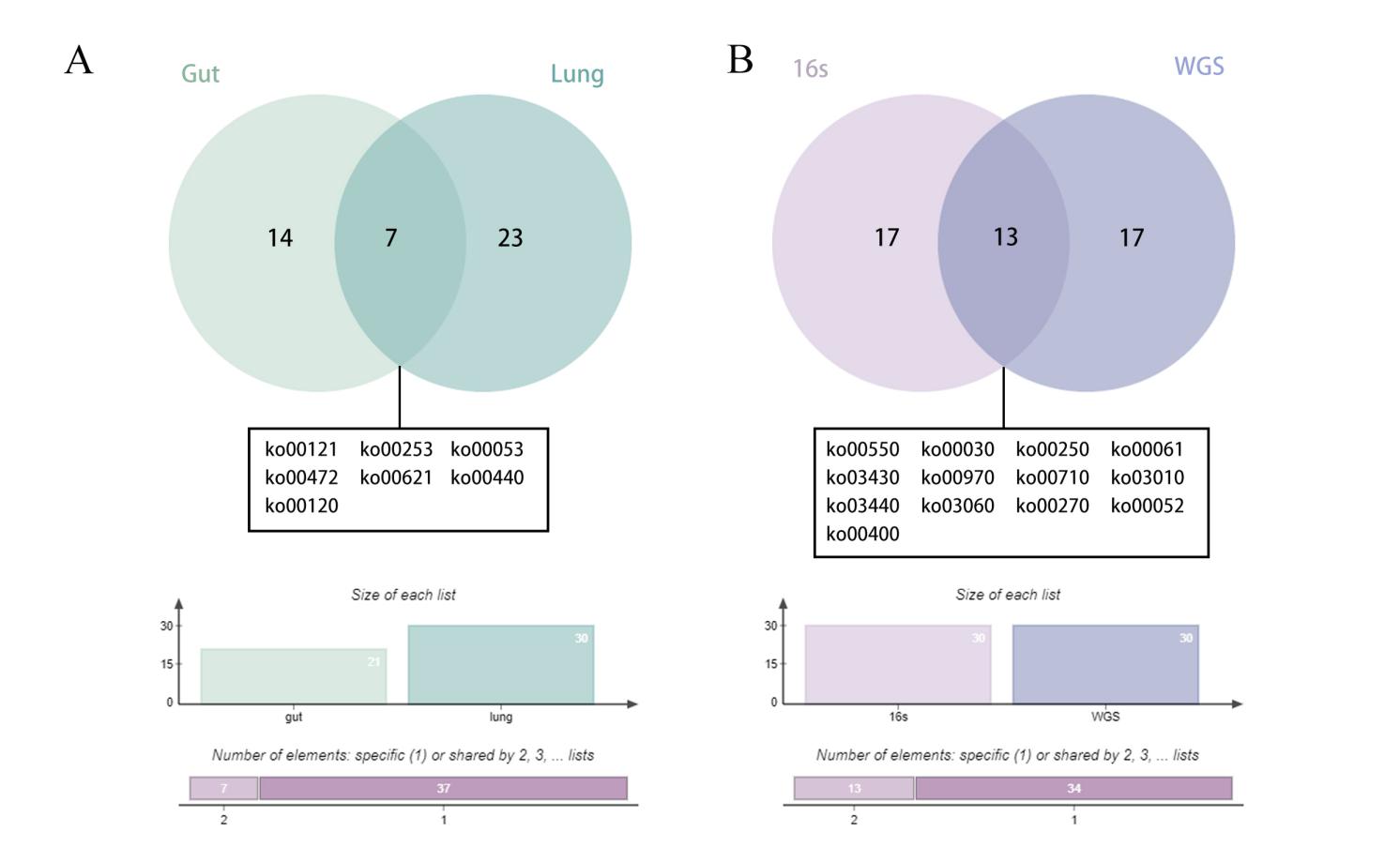


**Supplementary Figure 6.** (A) The signature pathways enriched in both gut and lung in the LC population. (B) Gut metagenomic samples were used to predict GM function related to LC and verify the results of 16s function prediction. LC, lung cancer; GM, gut microbiota.

# Supplementary Tables

**Supplementary Table 1.** Quality assessment results of the included studies with the ROBIS tool.

| **Review** | | | **Phase 1** | | | | **Phase 2** |
| --- | --- | --- | --- | --- | --- | --- | --- |
| **Disorder** | **Year** | **First Author** | **Study  eligibility  criteria** | **Identification and  selection of studies** | **Data collection  and study appraisal** | **Synthesis  and findings** | **RISK OF BIAS IN THE  REVIEW*** |
| NSCLC and DM | 2021 | Hui Lu | low | low | low | low | low |
| LC | 2021 | Feng Zhao | low | low | low | low | low |
| NSCLC and DM | 2021 | Se-Hoon Lee | low | low | unclear | low | low |
| LC | 2019 | He Zhuang | low | low | low | low | low |
| LC, ILD and COPD | 2021 | Susana Seixas | high | unclear | high | unclear | high |
| LC | 2018 | Jun-Chieh J. Tsay | low | unclear | low | low | low |
| COPD | 2021 | Mohammadali Yavari Ramsheh | high | low | low | low | low |
| LC | 2020 | Yoshitaro Heshiki | high | high | low | high | high |
| TB | 2021 | Demin Cao | low | low | low | unclear | low |
| Asthma | 2018 | Lesa Begley | unclear | low | unclear | unclear | unclear |
| LC, ILD and COPD | 2021 | Susana Seixas | low | low | high | unclear | unclear |
| COPD and ILD | 2021 | Shashank Gupta | low | low | unclear | low | low |
| IPF and CHP | 2020 | Rachele Invernizzi | unclear | high | unclear | unclear | high |

Low = low risk of bias, high = high risk of bias, unclear = insufficient information to assess the risk of bias; *this criterion is scored by answering the following questions: A. Did the interpretation of the findings address all of the concerns identified in the Phase 2 assessment?; B. Was the relevance of identified studies to the review's research question appropriately considered?; and C. Did the reviewers avoid emphasizing results based on their statistical significance. NSCLC, non-small cell lung cancer; DM, distant metastasis; LC, lung cancer; ILD, interstitial lung disease; COPD, chronic obstructive pulmonary disease; TB, tuberculosis; IPF, idiopathic pulmonary fibrosis; CHP, Chronic hypersensitivity pneumonitis.

**Summary:** The risk of bias in the systematic reviews was low (Supplementary Table 1). The primary concern was the lack of consideration of confounding factors in some of the studies. This led us to fail to assess some factors in our analysis (e.g., age, gender, psychotropic medication) as other, particularly lifestyle factors such as diet.

**Supplementary Table 2.** Quality assessment results of the included studies using the Joanna Briggs Institute Critical Appraisal Checklist for Case-Control Studies.

| **Study(First Author & Year)** | **1. Were the criteria for inclusion in the sample clearly defined?** | **2. Were the study subjects and the setting described in detail？** | **3. Was the exposure measured in a valid and reliable way？** | **4. Were objective, standard criteria used for measurement of the condition?** | **5. Were confounding factors identified?** | **6. Were the outcomes measured in a valid and reliable way?** | **7. Were the outcomes measured in a valid and reliable way?** | **8. Was appropriate statistical analysis used?** |
| --- | --- | --- | --- | --- | --- | --- | --- | --- |
| Hui Lu, 2021 | Yes | Yes | Yes | Yes | Yes | Yes | Yes | Yes |
| Feng Zhao, 2021 | Yes | Yes | Yes | Yes | Yes | Yes | Yes | Yes |
| Se-Hoon Lee, 2021 | Yes | Yes | Yes | Yes | NO | Yes | Yes | Yes |
| He Zhuang, 2019 | Yes | Yes | Yes | Yes | Yes | Yes | Yes | Yes |
| Susana Seixas, 2021 | Yes | Yes | Yes | Yes | Yes | Yes | Yes | Yes |
| Jun-Chieh J. Tsay, 2018 | Yes | Yes | Yes | Yes | Yes | Yes | Yes | Yes |
| Mohammadali Yavari Ramsheh, 2021 | Yes | Yes | Yes | Yes | Yes | Yes | Yes | Yes |
| Yoshitaro Heshiki, 2020 | Yes | Yes | Yes | Yes | NO | Yes | Yes | Yes |
| Demin Cao, 2021 | Yes | Yes | Yes | Yes | NO | Yes | Yes | Yes |
| Lesa Begley, 2018 | Yes | Yes | Yes | Yes | Yes | Yes | Yes | Yes |
| Susana Seixas, 2021 | Yes | Yes | Yes | Yes | Yes | Yes | Yes | Yes |
| Shashank Gupta, 2021 | Yes | Yes | Yes | Yes | Yes | Yes | Yes | Yes |
| Rachele Invernizzi, 2020 | Yes | Yes | Yes | Yes | Yes | Yes | Yes | Yes |

##

.

**Supplementary Table 3.** Methodology of sample ( gut and lung) processing of the included studies

| **Study** | **Sample type** | **Collection method** | **Long-term storage condition** | **DNA extraction method** | **primer set** |
| --- | --- | --- | --- | --- | --- |
| Lu et al.2021 | Fecal | - | immediately stored at −80°C | using the OMEGA-soil DNA kit (Omega Bio-Tek, USA) | 338F (5′-ACTCCTACGGGAGGCAGCAG-3′) and 806R (5′-GGACTACHVGGGTWTCTAAT-3′) |
| Zhao et al.2021 | Fecal | Stool samples were collected in the morning after overnight fasting (≥ 8h). The stool samples were divided into five equal parts (each 200mg) and put in sterile frozen pipes. | storage at -80°C | using the E.Z.N.A. Stool DNA Kit (Omega, USA), | 341F: 5’-CCTACGGGNGGCWGCAG-3’ and 805R: 5’- GACTACHVGGGTATCTAATCC -3’ |
| Lee et al.2021 | Fecal | - | immediately stored in the freezer (−20 °C) for  One day and transferred in a provided cooler to our facilities, where they were stored.  at −80 °C | using a FastDNA SPIN kit for soil (MP Biomedicals) | forward, 5 ′- TC GT CG GC AG CG TC AG AT GT GT AT AA GA GA CA GC CTACGGGNGGCWGCAG-3′; reverse, 5′-GTCTCGTGGGCTCGGAGATGTGTATAAGAGAC  AGGACTACHVGGGTATCTAATCC-3′. |
| Zhuang et al., 2019 | Fecal | frozen in liquid nitrogen | stored in a −80°C freezer | the OMG-soil kit (Omega Bio-tek, Norcross, GA, USA). | 338F (5′-ACTCCTACGGGAGGCAGCAG-3′) and 806R (5′-GGACTACHVGGGTWTCTAAT-3′) |
| Seixas et al., 2021 | BALF | bronchoscopy | stored by pulmonologists at − 20 to 4 °C and then transported on ice to research centers where they were stored at − 80 °C until needed | using DNA Mini kit (Qiagen) | - |
| J. Tsay et al., 2018 | BALF | underwent clinical bronchoscopy | - | - | - |
| Ramsheh et al., 2021 | bronchial brushes | underwent video­assisted bronchoscopy | - | the AllPrep DNA/RNA Mini Kit (Qiagen,  Hilden, Germany) | - |
| Heshiki et al., 2020 | Fecal | - | - | Library preparation (using KAPA Hyper Prep Kit KR0961-V1.14) and Illumina sequencing were done at the University of Hong Kong, | - |
| Demin et al..2021 | Fecal | - | stored at −80°C | using the QIAamp DNA stool minikit | the F3 (5′-CCTACGGGNBGCASCAG-3′) and R4 (5′-GACTACNVGGGTATCTAATCC-3′) primers |
| Lesa et al., 2018 | Fecal | using a commercial kit containing a DNA stabilization reagent (OMR-200; DNA Genotek) | - | The total DNA was extracted using a cetyltrimethylammonium bromide buffer-based protocol coupled with bead-beating | - |
| Susana et al.2021 | BALF | bronchoscopy | stored at − 80 °C until needed | using DNA Mini kit (Qiagen) | - |
| Shashank et al., 2021 | BALF | bronchoscopy | - | with Qubit 2.0 using high sensitivity DNA quantification kit (Invitrogen, USA) | Fwd 5′-TCGTCGGCAGCGTCAGATGTGTATAAGAGACAGCCTACGGGNGGCWGCAG-3` and Rev 5′-GTCTCGTGGGCTCGGAGATGTGTATAAGAGACAGGACTACHVGGGTATCTA ATCC-3`) |
| Rachele et al., 2020 | BALF | bronchoscopy |  | Genomic DNA was extracted from BAL cell pellets as previously described, with minor modifications | using the barcoded universal primer pair 515F/806R |

**Supplementary Table 4.** Methodology and findings of studying beta diversity in included studies

| **Disorder** | **Study** | **Year** | **Metric** | **Analysis** | **Test** | **Finding** |
| --- | --- | --- | --- | --- | --- | --- |
| LC | Hui Lu | 2021 | Bray-Curtis | PCoA | Adonis test | Sig. different |
| LC | Feng Zhao | 2021 | Bray-Curtis; Unweighted Unifrac | PCoA | ANOSIM test | Sig. different |
| LC | Se-Hoon Lee | 2021 | Unweighted Unifrac | PCoA | Kruskal–Wallis test | Sig. different |
| LC | He Zhuang | 2019 | - | NMDS | - | Sig. different |
| LC; COPD; ILD | Susana Seixas | 2021 | unweighted and weighted UniFrac, Bray–Curtis and Jaccard distances | PCoA | Adonis test | No sig. different |
| LC; Benign; HC | Jun-Chieh J. Tsay | 2018 | Bray-Curtis | PERMANOVA | the Mann-Whitney test or the Kruskal-Wallis ANOVA. | Sig. different |
| COPD | Mohammadali Yavari Ramsheh | 2021 | unweighted and weighted UniFrac | permutation multivariate analysis of variance |  | No sig. different |
| LC | Yoshitaro Heshiki | 2020 | Bray-Curtis | NMDS | ANOSIM test | Sig. different |
| TB | Demin Cao | 2021 | unweighted and weighted UniFrac | PCoA | Adonis test | Sig. different |
| Asthma | Lesa Begley | 2018 | Bray-Curtis;Unifrac | PERMANOVA | Wilcoxon rank-sum or Kruskal-Wallis | Sig. different |
| LC; COPD; ILD | Susana Seixas | 2021 | unweighted and weighted UniFrac, Bray–Curtis and Jaccard distances | PCoA | Adonis test | No sig. different |
| COPD; ILD | Shashank Gupta | 2021 | Bray-Curtis | PCA | - | No sig. different |
| CHP; IPF | Rachele Invernizzi | 2020 | Euclidean | PCA; PERMANOVA | - | Sig. different |

LC, lung cancer; ILD, interstitial lung disease; COPD, chronic obstructive pulmonary disease; TB, tuberculosis; IPF, idiopathic pulmonary fibrosis; CHP, Chronic hypersensitivity pneumonitis; PCoA, Principle coordinate analysis; NMDS, non-metric multidimensional scaling; PERMANOVA, permutational analysis of variance; PCA, Principal Components Analysis.

**Supplementary Table 5.** Microbial biomarkers in gut microbiota associated with lung cancer.

| Biomarker_names | Logarithm_value | Groups | LDA_value | P_value |
| --- | --- | --- | --- | --- |
| g__Bifidobacterium | 4.013187322 | Lung_cancer | 2.842784659 | 0.046425379 |
| g__Collinsella | 3.279417601 | Lung_cancer | 2.51501191 | 0.006211577 |
| g__Prevotella | 4.104176741 | Normal | 3.643459764 | 9.63E-06 |
| g__Enterococcus | 3.791738284 | Lung_cancer | 3.504641742 | 8.63E-07 |
| g__Lactobacillus | 3.737573739 | Lung_cancer | 3.232045605 | 4.49E-07 |
| g__Streptococcus | 3.725280525 | Lung_cancer | 3.117907476 | 0.018201291 |
| g__Blautia | 4.215173679 | Normal | 2.948693917 | 5.75E-05 |
| g__Coprococcus | 3.94137089 | Normal | 3.301158464 | 1.04E-09 |
| g__Dorea | 3.36109406 | Lung_cancer | 2.506118847 | 0.002698908 |
| g__Roseburia | 4.039536886 | Normal | 2.961835167 | 6.41E-05 |
| g__Faecalibacterium | 4.395814489 | Normal | 3.394807187 | 5.62E-06 |
| g__Megamonas | 3.589727054 | Normal | 2.999375904 | 0.001701603 |
| g___Eubacterium_ | 3.23175331 | Normal | 2.609607816 | 0.000830748 |
| g__Catenibacterium | 3.436496939 | Normal | 2.778560023 | 0.000537524 |
| g__Escherichia | 4.185578144 | Lung_cancer | 3.211940398 | 0.013158944 |
| g__Klebsiella | 3.558193938 | Lung_cancer | 3.015288761 | 0.016604248 |

**Supplementary Table 6.** Microbial biomarkers in lung microbiota associated with lung cancer.

| Biomarker_names | Logarithm_value | Groups | LDA_value | P_value |
| --- | --- | --- | --- | --- |
| g__Actinomyces | 4.374811292 | Benign | 3.966774173 | 0.004095891 |
| g__Rothia | 4.184826924 | Benign | 3.80354848 | 0.002470479 |
| g__Atopobium | 3.334289963 | Normal | 2.936716072 | 1.32E-08 |
| g__Porphyromonas | 4.40212929 | Normal | 3.885162658 | 3.69E-06 |
| g__Prevotella | 5.571205388 | Normal | 5.089317209 | 4.14E-07 |
| g__Bacillus | 4.75865442 | Lung_cancer | 4.442820482 | 5.33E-11 |
| g__Listeria | 4.544721831 | Lung_cancer | 4.236306524 | 2.27E-12 |
| g__Staphylococcus | 4.854274957 | Lung_cancer | 4.517891535 | 6.57E-08 |
| g__Granulicatella | 3.973618035 | Lung_cancer | 3.417363973 | 0.035285915 |
| g__Enterococcus | 3.91560395 | Lung_cancer | 3.608612933 | 2.96E-12 |
| g__Vagococcus | 3.499082941 | Benign | 3.139988302 | 1.43E-06 |
| g__Lactobacillus | 4.70085449 | Lung_cancer | 4.393675055 | 1.64E-10 |
| g__Megasphaera | 3.813793162 | Lung_cancer | 3.510982228 | 3.49E-05 |
| g__Selenomonas | 3.865774254 | Benign | 3.461154905 | 0.000532604 |
| g__Fusobacterium | 4.309143492 | Normal | 3.922976967 | 9.33E-10 |
| g__Leptotrichia | 4.219728904 | Normal | 3.815301894 | 6.78E-07 |
| g__Methylobacterium | 4.084369658 | Lung_cancer | 3.788223943 | 0.000768853 |
| g__Polaromonas | 3.474743324 | Benign | 3.042605923 | 0.002433937 |
| g__Campylobacter | 3.796079033 | Normal | 3.385450843 | 5.61E-07 |
| g__Shewanella | 4.492346167 | Normal | 4.21017236 | 1.42E-23 |
| g__Escherichia | 4.934351165 | Lung_cancer | 4.632754212 | 6.92E-12 |
| g__Klebsiella | 3.748548484 | Benign | 3.389416005 | 0.000977922 |
| g__Halomonas | 4.918124625 | Normal | 4.633499288 | 3.17E-24 |
| g__Actinobacillus | 3.858404806 | Benign | 3.528174534 | 0.000218224 |
| g__Aggregatibacter | 3.649049919 | Lung_cancer | 3.025112303 | 0.012580293 |
| g__Haemophilus | 4.454282911 | Benign | 3.910658882 | 0.001585801 |
| g__Enhydrobacter | 3.331656933 | Lung_cancer | 3.072380997 | 0.013194768 |
| g__Moraxella | 4.234993575 | Benign | 4.017815741 | 0.001438184 |
| g__Pseudomonas | 4.466150491 | Benign | 4.150201992 | 4.54E-11 |

**Supplementary Table 7.** Functional difference of gut microbiota between the Lung cancer group and the Normal group.

| Biomarker_names | Logarithm_value | Groups | LDA_value | P_value | description |
| --- | --- | --- | --- | --- | --- |
| ko00020 | 3.945480858 | Lung_cancer | 2.146360785 | 0.020501708 | Citrate cycle (TCA cycle) |
| ko00053 | 3.491983325 | Lung_cancer | 2.319430873 | 0.001420191 | Ascorbate and aldarate metabolism |
| ko00120 | 3.183345727 | Lung_cancer | 2.00238631 | 6.94E-05 | Primary bile acid biosynthesis |
| ko00121 | 4.136945182 | Lung_cancer | 2.950011458 | 7.45E-05 | Secondary bile acid biosynthesis |
| ko00130 | 3.497431615 | Lung_cancer | 2.37850742 | 0.000549753 | Ubiquinone and other terpenoid-quinone biosynthesis |
| ko00253 | 3.334623404 | Lung_cancer | 2.904978662 | 6.64E-10 | Tetracycline biosynthesis |
| ko00281 | 2.968697117 | Lung_cancer | 2.036447486 | 0.040335813 | Geraniol degradation |
| ko00350 | 3.438257816 | Lung_cancer | 2.029579822 | 1.50E-06 | Tyrosine metabolism |
| ko00430 | 3.816248403 | Lung_cancer | 2.358263404 | 1.57E-09 | Taurine and hypotaurine metabolism |
| ko00440 | 3.297964065 | Lung_cancer | 2.053067944 | 0.000156069 | Phosphonate and phosphinate metabolism |
| ko00450 | 4.029521286 | Lung_cancer | 2.088675943 | 0.000195685 | Selenocompound metabolism |
| ko00460 | 3.57691614 | Lung_cancer | 2.708477166 | 0.027603517 | Cyanoamino acid metabolism |
| ko00472 | 3.300589254 | Lung_cancer | 2.232790856 | 0.00213556 | D-Arginine and D-ornithine metabolism |
| ko00480 | 3.694506013 | Lung_cancer | 2.430390761 | 8.02E-07 | Glutathione metabolism |
| ko00520 | 4.079738537 | Lung_cancer | 2.185220278 | 0.005304816 | Amino sugar and nucleotide sugar metabolism |
| ko00621 | 3.403749808 | Lung_cancer | 2.129492874 | 0.012921073 | Dioxin degradation |
| ko00623 | 3.167342585 | Lung_cancer | 2.441875808 | 0.000851447 | Toluene degradation |
| ko00627 | 3.093431365 | Lung_cancer | 2.139439562 | 9.46E-12 | Aminobenzoate degradation |
| ko00642 | 2.937580537 | Lung_cancer | 2.442258243 | 6.26E-07 | Ethylbenzene degradation |
| ko00785 | 3.84480045 | Lung_cancer | 2.817548431 | 4.62E-06 | Lipoic acid metabolism |
| ko00980 | 2.665680663 | Lung_cancer | 2.262499829 | 2.73E-07 | Metabolism of xenobiotics by cytochrome P450 |
| ko00250 | 4.250462326 | Normal | 2.3617514 | 0.000113482 | Alanine, aspartate and glutamate metabolism |
| ko00270 | 4.135659715 | Normal | 2.119210347 | 0.005541504 | Cysteine and methionine metabolism |
| ko00290 | 4.36671463 | Normal | 2.574669541 | 0.000385296 | Valine, leucine, and isoleucine biosynthesis |
| ko00300 | 4.23330826 | Normal | 2.272829083 | 0.007835315 | Lysine biosynthesis |
| ko00330 | 3.88975739 | Normal | 2.029468812 | 4.44E-05 | Arginine and proline metabolism |
| ko00340 | 4.119991555 | Normal | 2.55760225 | 3.59E-05 | Histidine metabolism |
| ko00400 | 4.108694327 | Normal | 2.555616468 | 2.44E-10 | Phenylalanine, tyrosine, and tryptophan biosynthesis |
| ko00410 | 3.644520606 | Normal | 2.158797639 | 0.008733225 | beta-Alanine metabolism |
| ko00471 | 4.329044392 | Normal | 2.387943377 | 0.013161653 | D-Glutamine and D-glutamate metabolism |
| ko00524 | 3.280921428 | Normal | 2.136688232 | 0.03580776 | Butirosin and neomycin biosynthesis |
| ko00550 | 4.292311612 | Normal | 2.425977622 | 0.003655647 | Peptidoglycan biosynthesis |
| ko00660 | 4.261588064 | Normal | 2.274656879 | 0.007035525 | C5-Branched dibasic acid metabolism |
| ko00670 | 4.241957961 | Normal | 2.294728948 | 9.68E-05 | One carbon pool by folate |
| ko00710 | 4.208983308 | Normal | 2.246509208 | 6.72E-05 | Carbon fixation in photosynthetic organisms |
| ko00730 | 4.247531157 | Normal | 2.702760427 | 1.57E-10 | Thiamine metabolism |
| ko00740 | 3.954549756 | Normal | 2.504369523 | 3.46E-08 | Riboflavin metabolism |
| ko00760 | 4.053461839 | Normal | 2.256544404 | 1.65E-07 | Nicotinate and nicotinamide metabolism |
| ko00770 | 4.265516989 | Normal | 2.484919276 | 1.72E-07 | Pantothenate and CoA biosynthesis |
| ko00860 | 3.87078778 | Normal | 2.426484326 | 4.13E-05 | Porphyrin and chlorophyll metabolism |
| ko00900 | 4.119047999 | Normal | 2.265657865 | 0.000180759 | Terpenoid backbone biosynthesis |
| ko01051 | 4.72286505 | Normal | 3.114019967 | 0.001093721 | Biosynthesis of ansamycins |
| ko02030 | 4.060147864 | Normal | 2.956806339 | 5.53E-08 | Bacterial chemotaxis |
| ko02040 | 3.825243825 | Normal | 2.782864388 | 1.18E-06 | Flagellar assembly |
| ko03020 | 4.076209547 | Normal | 2.681915724 | 5.82E-09 | RNA polymerase |
| ko03060 | 4.159479903 | Normal | 2.091587165 | 0.008109168 | Protein export |
| ko03430 | 4.230765152 | Normal | 2.204174339 | 0.003515158 | Mismatch repair |
| ko03440 | 4.183132664 | Normal | 2.271904786 | 8.77E-05 | Homologous recombination |
| ko04112 | 4.18345844 | Normal | 2.325487571 | 6.14E-06 | Cell cycle - Caulobacter |
| ko04122 | 4.066761669 | Normal | 2.402990403 | 0.000193202 | Sulfur relay system |

**Supplementary Table 8.** Functional difference of lung microbiota between the Lung cancer group and the Normal group.

| Biomarker_names | Logarithm_value | Groups | LDA_value | P_value | description |
| --- | --- | --- | --- | --- | --- |
| ko00196 | 2.499602078 | Benign | 2.200418761 | 0.029920113 | Photosynthesis - antenna proteins |
| ko00362 | 3.390903607 | Benign | 2.546917606 | 8.98E-05 | Benzoate degradation |
| ko00363 | 3.194817066 | Benign | 2.581779858 | 0.013251425 | Bisphenol degradation |
| ko00364 | 2.928287695 | Benign | 2.44977613 | 5.58E-05 | Fluorobenzoate degradation |
| ko00460 | 3.72852762 | Benign | 2.772285128 | 0.037605587 | Cyanoamino acid metabolism |
| ko00562 | 3.480152687 | Benign | 2.579278123 | 0.000731211 | Inositol phosphate metabolism |
| ko00564 | 3.819925926 | Benign | 2.341115051 | 0.000154101 | Glycerophospholipid metabolism |
| ko00627 | 3.360682559 | Benign | 2.484661258 | 0.000816885 | Aminobenzoate degradation |
| ko00643 | 3.330164795 | Benign | 2.536706812 | 0.012275082 | Styrene degradation |
| ko00660 | 4.242956212 | Benign | 3.022987423 | 0.00012011 | C5-Branched dibasic acid metabolism |
| ko00910 | 3.865187 | Benign | 2.359123896 | 0.038142336 | Nitrogen metabolism |
| ko01051 | 4.573418171 | Benign | 3.517281111 | 1.53E-06 | Biosynthesis of ansamycins |
| ko02010 | 3.919223354 | Benign | 2.986557768 | 6.55E-09 | ABC transporters |
| ko00020 | 4.104647753 | Normal | 2.676299594 | 0.000147598 | Citrate cycle (TCA cycle) |
| ko00140 | 2.455866846 | Normal | 2.071670323 | 4.38E-13 | Steroid hormone biosynthesis |
| ko00190 | 3.747880069 | Normal | 2.676279143 | 4.14E-11 | Oxidative phosphorylation |
| ko00195 | 3.513918495 | Normal | 2.915593548 | 0.005105993 | Photosynthesis |
| ko00250 | 4.178518357 | Normal | 2.682812234 | 0.000318847 | Alanine, aspartate and glutamate metabolism |
| ko00510 | 2.914528477 | Normal | 2.039854509 | 0.000568127 | N-Glycan biosynthesis |
| ko00511 | 4.121605779 | Normal | 3.4110617 | 7.88E-08 | Other glycan degradation |
| ko00521 | 4.172919578 | Normal | 3.051569695 | 2.30E-07 | Streptomycin biosynthesis |
| ko00531 | 3.708404203 | Normal | 3.153137464 | 1.20E-09 | Glycosaminoglycan degradation |
| ko00540 | 4.228496684 | Normal | 3.517027097 | 1.07E-06 | Lipopolysaccharide biosynthesis |
| ko00550 | 4.293898729 | Normal | 2.907947752 | 0.017648058 | Peptidoglycan biosynthesis |
| ko00600 | 3.507983489 | Normal | 2.757182156 | 1.12E-08 | Sphingolipid metabolism |
| ko00623 | 3.593877638 | Normal | 3.070167137 | 2.70E-05 | Toluene degradation |
| ko00670 | 4.287371227 | Normal | 3.160024506 | 2.46E-05 | One carbon pool by folate |
| ko00720 | 4.093573378 | Normal | 2.804780388 | 7.24E-09 | Carbon fixation pathways in prokaryotes |
| ko00740 | 4.026904542 | Normal | 2.950753705 | 0.000323771 | Riboflavin metabolism |
| ko00750 | 4.096636806 | Normal | 2.760244093 | 9.61E-07 | Vitamin B6 metabolism |
| ko00760 | 4.097976288 | Normal | 3.016094031 | 1.01E-09 | Nicotinate and nicotinamide metabolism |
| ko00790 | 4.220493454 | Normal | 2.890592032 | 0.00078507 | Folate biosynthesis |
| ko00908 | 3.907318473 | Normal | 2.982367852 | 0.000215039 | Zeatin biosynthesis |
| ko00960 | 3.676455632 | Normal | 2.970703553 | 2.51E-06 | Tropane, piperidine, and pyridine alkaloid biosynthesis |
| ko00980 | 3.391495079 | Normal | 2.813736894 | 0.047533405 | Metabolism of xenobiotics by cytochrome P450 |
| ko00983 | 4.158937663 | Normal | 3.327883514 | 1.19E-09 | Drug metabolism - other enzymes |
| ko01055 | 4.294946621 | Normal | 3.422233828 | 1.36E-11 | Biosynthesis of vancomycin group antibiotics |
| ko03020 | 3.956001632 | Normal | 2.883669407 | 8.44E-11 | RNA polymerase |
| ko03410 | 4.032050066 | Normal | 2.564640994 | 0.001413895 | Base excision repair |
| ko04112 | 4.202991178 | Normal | 2.916319878 | 0.002536644 | Cell cycle - Caulobacter |
| ko04210 | 2.889293332 | Normal | 2.291416989 | 1.46E-06 | Apoptosis |
| ko04621 | 3.001038248 | Normal | 2.329985682 | 1.54E-10 | NOD-like receptor signaling pathway |
| ko04626 | 3.32681936 | Normal | 2.101759007 | 3.39E-06 | Plant-pathogen interaction |
| ko04974 | 2.912537099 | Normal | 2.38970447 | 6.51E-07 | Protein digestion and absorption |
| ko05111 | 3.422106558 | Normal | 2.683591234 | 3.26E-11 | Vibrio cholerae pathogenic cycle |
| ko00030 | 4.209981412 | Lung_cancer | 2.987905913 | 5.98E-14 | Pentose phosphate pathway |
| ko00040 | 3.81226389 | Lung_cancer | 3.094363113 | 0.000105807 | Pentose and glucuronate interconversions |
| ko00051 | 4.003667477 | Lung_cancer | 2.927859762 | 1.69E-06 | Fructose and mannose metabolism |
| ko00052 | 3.95855525 | Lung_cancer | 2.75784794 | 0.000183231 | Galactose metabolism |
| ko00053 | 3.66764752 | Lung_cancer | 2.86156056 | 1.09E-06 | Ascorbate and aldarate metabolism |
| ko00061 | 4.253043821 | Lung_cancer | 3.04144982 | 1.87E-06 | Fatty acid biosynthesis |
| ko00120 | 2.858424873 | Lung_cancer | 2.490221641 | 8.30E-05 | Primary bile acid biosynthesis |
| ko00121 | 3.793393145 | Lung_cancer | 3.430061675 | 0.000142783 | Secondary bile acid biosynthesis |
| ko00253 | 3.687837601 | Lung_cancer | 3.137583605 | 0.001663954 | Tetracycline biosynthesis |
| ko00311 | 3.159773858 | Lung_cancer | 2.320703151 | 0.002065274 | Penicillin and cephalosporin biosynthesis |
| ko00312 | 3.336856395 | Lung_cancer | 2.796996963 | 5.27E-05 | beta-Lactam resistance |
| ko00440 | 3.338606331 | Lung_cancer | 2.529220082 | 5.34E-12 | Phosphonate and phosphinate metabolism |
| ko00472 | 3.837515602 | Lung_cancer | 3.347839287 | 5.31E-12 | D-Arginine and D-ornithine metabolism |
| ko00473 | 4.317428089 | Lung_cancer | 3.269934119 | 2.13E-12 | D-Alanine metabolism |
| ko00500 | 3.914676456 | Lung_cancer | 2.524615285 | 0.014696278 | Starch and sucrose metabolism |
| ko00561 | 3.722989965 | Lung_cancer | 2.904650673 | 3.53E-12 | Glycerolipid metabolism |
| ko00620 | 4.121170195 | Lung_cancer | 3.023932491 | 2.55E-08 | Pyruvate metabolism |
| ko00621 | 3.424258259 | Lung_cancer | 3.086574843 | 2.55E-17 | Dioxin degradation |
| ko00633 | 3.349459485 | Lung_cancer | 2.490314787 | 0.000576604 | Nitrotoluene degradation |
| ko00730 | 4.184864987 | Lung_cancer | 2.844614654 | 0.000257788 | Thiamine metabolism |
| ko00906 | 2.978248926 | Lung_cancer | 2.570383604 | 4.42E-09 | Carotenoid biosynthesis |
| ko00920 | 3.999271628 | Lung_cancer | 2.620650264 | 0.001336726 | Sulfur metabolism |
| ko01053 | 3.267147232 | Lung_cancer | 2.634895268 | 6.88E-07 | Biosynthesis of siderophore group nonribosomal peptides |
| ko02020 | 3.651088037 | Lung_cancer | 2.691023719 | 0.021005275 | Two-component system |
| ko02060 | 3.913324626 | Lung_cancer | 3.432595026 | 4.55E-14 | Phosphotransferase system (PTS) |
| ko03450 | 2.663489721 | Lung_cancer | 2.291245753 | 3.39E-05 | Non-homologous end-joining |
| ko04122 | 4.086545027 | Lung_cancer | 3.249411257 | 8.54E-08 | Sulfur relay system |
| ko05100 | 2.749009385 | Lung_cancer | 2.384102962 | 4.37E-10 | Bacterial invasion of epithelial cells |
| ko05131 | 2.564375109 | Lung_cancer | 2.249974753 | 5.62E-12 | Shigellosis |
| ko05150 | 3.376052767 | Lung_cancer | 3.021434429 | 1.20E-12 | Staphylococcus aureus infection |
